# Suppression of Ehrlich ascites tumor cell proliferation via G1 arrest induced by dietary nucleic acid-derived nucleosides

**DOI:** 10.1371/journal.pone.0305775

**Published:** 2024-07-18

**Authors:** Nahoko Shiomi, Mamia Furuta, Yutaro Sasaki, Isao Matsui-Yuasa, Keisuke Kiriyama, Mica Fujita, Keita Sutoh, Akiko Kojima-Yuasa

**Affiliations:** 1 Department of Food and Human Health Sciences, Graduate School of Human Life Science, Osaka City University, Osaka, Japan; 2 Department of Nutrition, Graduate School of Human Life and Ecology, Osaka Metropolitan University, Osaka, Japan; 3 Fordays Co., Ltd., Tokyo, Japan; 4 Fordays Nutritional Research Center, Tokyo University of Agriculture and Technology, Tokyo, Japan; University of Cambridge, UNITED KINGDOM

## Abstract

The nucleic acids found in food play a crucial role in maintaining various bodily functions. This study investigated the potential anticancer effects of dietary nucleic acids, an area that is still not fully understood. By utilizing an *in vivo* mouse model and an *in vitro* cell model, we discovered an anti-proliferative impact of RNA in both systems. DNA exhibited anti-proliferative effects in the mouse model, while this phenomenon wasn’t observed in the *in vitro* cell model using Ehrlich ascites tumor (EAT) cells. Conversely, DNA hydrolysate demonstrated distinct anti-proliferative effects in EAT cells, suggesting that nucleotides or nucleosides generated during nucleic acid digestion act as active constituents. Furthermore, we examined various nucleosides and two sodium-independent equilibrative nucleoside transporter inhibitors (ENTs), identifying guanosine and 2’-deoxyguanosine as pivotal in the anti-proliferative effect. We also found that the anti-proliferation activity with both nucleosides was suppressed by the treatment of dipyridamole, a non-selective inhibitor for ENT1 and ENT2, but not nitrobenzylthioinosine, a low inhibitor for ENT2. The uptake of these compounds into cells is likely facilitated by ENT2. These nucleotides impeded the progression of cancer cells from the G1 phase to the S phase in the cell cycle. Another significant finding is the increased expression of CCAAT/enhancer-binding protein (C/EBPβ) induced by guanosine and 2’-deoxyguanosine. Furthermore, immunostaining revealed that C/EBPβ diffuses into the nucleus, indicating its presence. This suggests that guanosine or 2-deoxyguanosine induces G1 arrest in cancer cells via the activation of C/EBPβ. Encouraged by these promising results, guanosine and 2’-deoxyguanosine show potential applications in cancer prevention.

## Introduction

Dietary nucleic acids have roles in maintaining various physiological functions in cells and organs. Their involvement in sustaining immune responses in both animal and human systems has long been recognized [1–3]. Deficiencies in dietary nucleic acids have notably been linked to reduced functionality of T-lymphocytes, an effect observed to be reversible upon adequate dietary RNA supplementation [4]. Furthermore, reports suggest that dietary RNA intake may alleviate adipose tissue inflammation and improve glucose tolerance in mice exposed to a high-fat diet [5]. Moreover, the ingestion of DNA sourced from salmon milt has shown promising effects in ameliorating alcohol-induced liver injury [6]. These findings collectively indicate the potential therapeutic effects and multifaceted benefits associated with dietary nucleic acids in immune modulation and metabolic health.

In recent years, there have been reports indicating the potential protective effects of guanosine, a nucleoside, against degenerative diseases and injuries. Numerous reports particularly focus on the modulatory role of guanosine within the central nervous system [7–9]. Lanznaster *et al*. have compiled a review discussing the therapeutic potential of guanosine in brain disorders in relation to these findings [10].

There haven’t been many reports on the anticancer effects of nucleic acids or nucleosides. Gessi *et al*. reported that adenosine controls cancer progression through adenosine receptors [11]. Additionally, Garozzo *et al*. reported that guanine inhibits the growth of lymphoma and melanoma via G-protein-coupled receptors [12]. Furthermore, it has been reported that guanosine and its derivatives enhance the effects of chemotherapy drugs like acriflavin [13] and 5’-deoxy-5-fluorouridine [14]. However, the effects of nucleic acids or guanosine on cancer, the world’s second leading cause of death, have yet to be clearly elucidated.

While guanosine has shown promising protective effects in various scenarios, the focus shifts in the present study to its impact on cancer-related processes. Recent investigations have highlighted the involvement of CCAAT/enhancer-binding protein (C/EBPβ) in suppressing proliferation and tumorigenesis while promoting differentiation [15]. Additionally, Long *et al*. demonstrated a reduction in C/EBPβ levels within cervical cancer tissue. They found that the overexpression of the C/EBPβ gene effectively restrained proliferation, invasion, and migration [16]. In this study, we examined the impact of guanosine, 2’-deoxyguanosine, and adenosine on the expression of C/EBPβ in Ehrlich ascites tumor (EAT) cells.

This study investigated the anti-proliferation effect of nucleic acids using both an *in vivo* mouse model and an *in vitro* cell model. ICR mice with EAT cells were selected as a suitable tumor model [17, 18] to elucidate the anti-proliferation activity of nucleic acids. EAT cells are fast-growing tumors characterized by increased progressive ascites fluid formation. Additionally, the study examined the effective structure of nucleic acid for anti-proliferative activity and its interaction with cells in an *in vitro* cell culture system using EAT cells.

## Materials and methods

### Materials

DNA, hydrolyzed DNA and RNA materials were provided from Fordays Co. Ltd. A DNA-rich fraction (DNA) and a hydrolyzed DNA-rich fraction (DNA hydrolysate) were prepared from Salmon milt. To prepare the DNA hydrolysate, DNA was hydrolyzed using a nuclease. The DNA-rich fraction consisted of DNA sodium (approximately 80%DNA). An RNA-rich fraction (RNA) was prepared from torula yeast (approximately 70% RNA).

### Animals

This study was conducted in compliance with the Guidelines for Proper Conduct of Animal Experiments of Science Council of Japan. The breeding and experiments involving mice were approved by the Ethics Committee of Laboratory Animals (Permission numbers: S0056 and S0097) and conducted in accordance with the animal experimentation regulations of Osaka City University.

**Fig 1:**
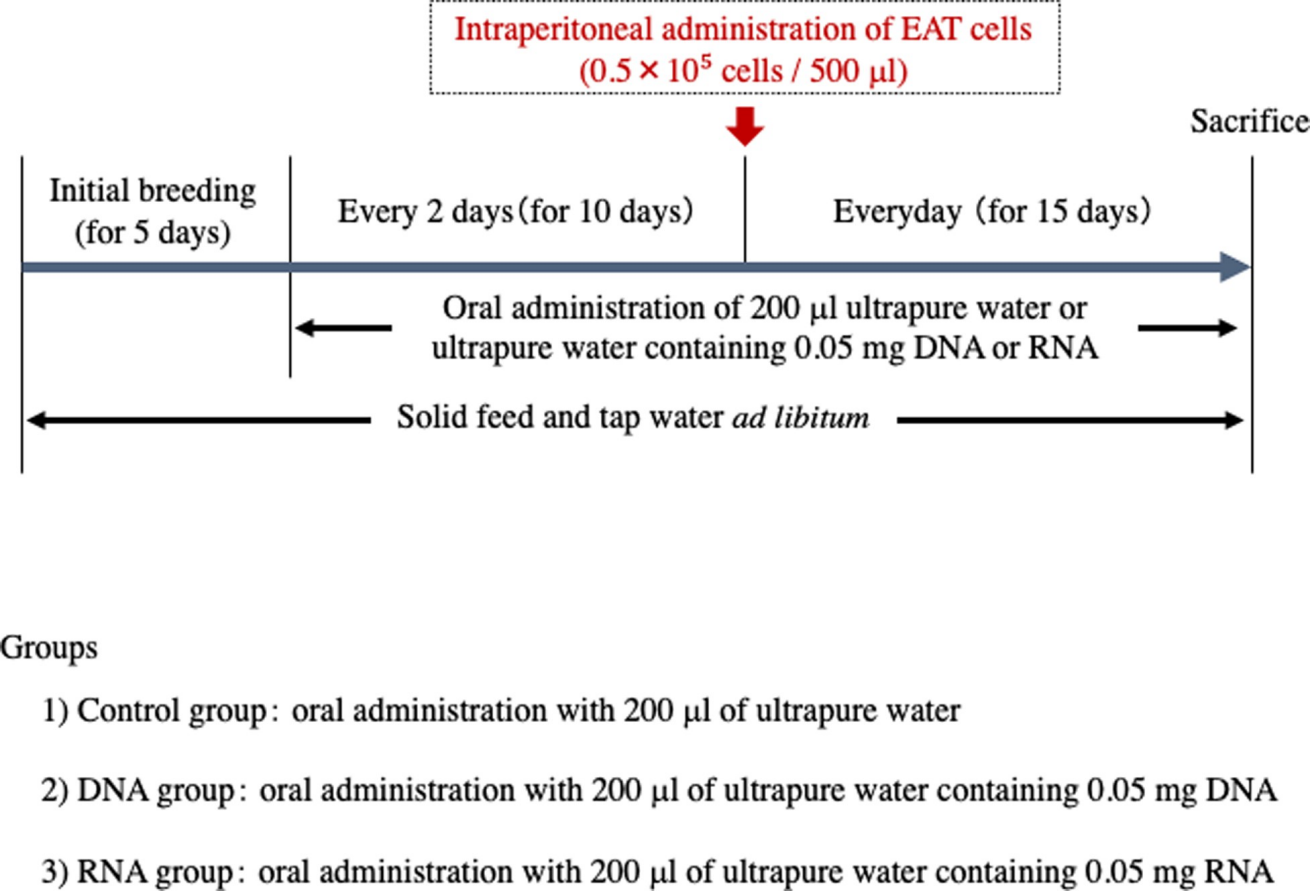
Experimental design.

ICR male mice weighing 28 to 30 g (6 weeks old) were obtained from Japan SLC, in Shizuoka, Japan. The mice were housed in groups of 3 per cage and provided with solid feed (laboratory MR stock) and tap water ad libitum until the end of the experiment. The room was illuminated from 8 am to 8 pm, and the temperature was maintained at 23 ± 1°C. After an initial breeding period of 5 days, the mice’s body weight was measured. Based on their weights, the mice were divided into the following three groups to ensure there was no bias in body weight, and the experiment commenced. The three groups were: a control group of mice administered intraperitoneally with 500 μl of EAT cell suspension adjusted to 1.0 x 10^5^ cells/ml, a DNA group of mice administered orally with 200 μl of ultrapure water containing 0.05 mg DNA every 2 days before the administration of EAT cells, and an RNA group of mice administered orally with 200 μl of ultrapure water containing 0.05 mg RNA every 2 days before the administration of EAT cells. The control group received only ultrapure water orally. Ten days after the experiment started, a suspension of EAT cells was intraperitoneally administered to the mice, with the samples continuing to be administered orally on a daily basis. Twenty-five days after the experiment commenced, the mice were dissected under deep isoflurane inhalation anesthesia to minimize pain, and the volume of ascites was measured. The experimental design was illustrated in Fig 1.

### Cell culture

EAT cells (JCRB9090) and 3T3-L1 cells (JCRB9014) were obtained from the Japanese Cancer Research Resource Bank (Tokyo, Japan), and cultured in Dulbecco’s Modified Eagle medium (DMEM) containing 10% fetal bovine serum and 0.1% penicillin/streptomycin in a humidified 5% CO_2_ incubator at 37°C.

### Measurement of cell number and cell viability of EAT

EAT cells (1 x 10^6^ cells/ml) were cultured for 24 h. Viable cells can excrete trypan blue from their cells, whereas dead cells cannot. By exploiting this difference, viable cells and dead cells can be distinguished. Cell viability and cell number were calculated by counting viable and dead cells using a Thoma hemacytometer.

### Measurement of cell viability of 3T3-L1

3T3-L1 cells (1 x 10^6^ cells/ml) were cultured for 24 h. Cell viability was assessed using a neutral red assay. After removing the medium, the 3T3-L1 cells were cultured with a neutral red solution for 2 h, followed by rinsing with a 1% formaldehyde and 1% calcium chloride solution. Subsequently, neutral red was extracted from the cells by treating them with a 1% acetic acid and 50% ethanol solution for 30 min. The absorbance of the extracted solution was measured at 540 nm using a spectrophotometer.

### Measurement of DNA synthesis

EAT cells (1 x 10^6^ cells/ml) were cultured in medium containing bromodeoxyuridine (BrdU) (FUJIFILM Wako Pure Chemical, Osaka, Japan) for 24 h and then fixed by adding 70% ethanol. After being denatured with 2N HCl for 30 min, the cells were incubated with 0.1 M tris-HCl (pH7.4) for 5 min and 0.1% Triton-X in PBS for 5 min. Subsequently the cells were then incubated with anti-BrdU antibody (DakoCytomation, Glostrup, Denmark), biotin-conjugated secondary antibody (DakoCytomation), and horseradish peroxidase-coupled streptavidin (DakoCytomation) for every 1 h. Finally, the cells were stained with 3,3’-diaminobenzidine tetrahydrochloride (DAB) dye as a substrate and BrdU-positive cells were observed using a microscope.

### Cell cycle analysis

To determine the cell cycle phases of samples, Muse^Ⓡ^ Cell Cycle Kit (MCH100106, Luminex Corporation, Austin, USA)was used according to the manufacturer’s protocol. EAT cells were collected by centrifugation at 300 x g for 5 min and washed once with PBS, then fixed with ice-cold 70% ethanol and incubated for at least 3 h at −20°C. 200 μl of the ethanol-fixed cell suspension was washed with 250 μl of PBS and further centrifuged at 300 x g for 5 min. Cell pellets were suspended in 200 μl of Muse^Ⓡ^ Cell Cycle Reagent, incubated for 30 mim at room temperature in the dark. The cell cycle was analyzed using Muse^Ⓡ^ Cell Analyzer(Merck KGaA, Darmastadt, Germany).

### Measurement of DNA content

The DNA contents of EAT cells were measured using the Burton procedure [19]. Briefly, cells were collected by centrifugation at 500 x g for 5 min and lysed with three cycles of freezing and thawing using 300 μl of 0.4 N perchloric acid. Subsequently, the lysate was centrifuged at 17400 x g for 20 min. The precipitate was then hydrolyzed with 600 μl of 0.4 N perchloric acid at 70°C, and centrifuged at 17400 x g for 15 min. The supernatant was utilized for DNA analysis. For each sample (0.4 mL), 0.1 mL of distilled water and 1 mL of DNA reagent (a mixture composed of a solution containing 1.5 g diphenylamine in 100 mL acetic acid and 1.5 mL H_2_SO_4_, combined with a solution containing 22.6 μL acetaldehyde in 1 mL distilled water, at a ratio of 200:1) were incubated at 36°C for 16 h. Absorbance at 600 nm was measured using a spectrophotometer.

### Quantitative reverse transcription-polymerase chain reaction (qRT-PCR)

Quantitative reverse transcription-polymerase chain reaction (qRT-PCR) was conducted to analyze the mRNA expression levels in EAT cells. Total RNA was extracted from the cells using the High Pure RNA Isolation Kit (Roche) and its quality and quantity were assessed with the 2100 Bioanalyzer (Agilent Technology). For cDNA synthesis, the PrimeScript RT Reagent Kit (TaKaRa Bio Inc.) was used. qRT-PCR was performed on the StepOnePlus PCR System (Thermo Fisher Scientific) using the TB Green Premix Ex Taq II (TaKaRa Bio Inc.). The PCR program consisted of initial denaturation at 95°C for 30 sec, followed by 40 cycles of denaturation at 95°C for 5 sec and annealing/extension at 60°C for 30 sec. The mRNA expression levels were normalized to β-actin. Delta-delta CT analysis was carried out using StepOne software v2.2.2 (Thermo Fisher Scientific). The primers used for C/EBPβ was F: 5’- AATCACTTAAAGATGTTCCTGCGG -3’, R; 5’- ATGCTCGAAACGGAAAAGGTTC -3’ and used for β-actin was F: 5’- GGAGATTACTGCCCTGGCTCCTA-3’, R; 5’- GACTCATCGTACTCCTGCTTGCTG -3’.

### Immunofluorescence microscopy

EAT cells were cultured for 16 h, and a portion of the cell suspension was applied onto a MAS-coat glass slide. The moisture was then completely removed by air drying at cold temperature. Subsequently, the microscope slide was washed twice with 0.5 ml of PBS. The slide was then immersed in methanol (-20°C) for 5 min for fixation. After being washed with PBS, the slide was immersed in 200 μl of PBS containing two drops of 0.1 M Triton X-100 for 5 min. After another wash with PBS, the slide was immersed in two drops of Protein Block Serum-free solution (DakoCytomation) for 30 min. Following another wash with PBS, the slide was incubated with the primary antibody, C/EBPβ antibody (Santa Cruz Biotechnology, Dallas, USA), and left at 4°C overnight. After washing with PBS, the microscope slide was treated with Alexa Fluor ^TM^ 488 goat anti-mouse IgG (H+L) (Thermo Fisher Scientific, Massachusetts, USA) for secondary antibody for 1 h. Following another wash with PBS, a cover glass was placed on the microscope slide with ProLong^TM^ Gold antifade regent (Thermo Fisher Scientific), and the slide was dried in the shade. The microscope slides were observed using a fluorescence microscope (LS101, Olympus Corporation, Tokyo, Japan).

### Statistical analysis

To compare multiple groups, we employed a one-way ANOVA followed by the Tukey-Kramer test. The significant difference test was conducted at a significance level of 5%, 1% or 0.1%. In the *in vivo* animal experiments, the data were presented as mean ± SE, while in the *in vitro* cell culture experiments, they were presented as mean ± SD.

## Results

### Anti-tumor activity of nucleic acids in EAT cells-bearing mice

**Fig 2:**
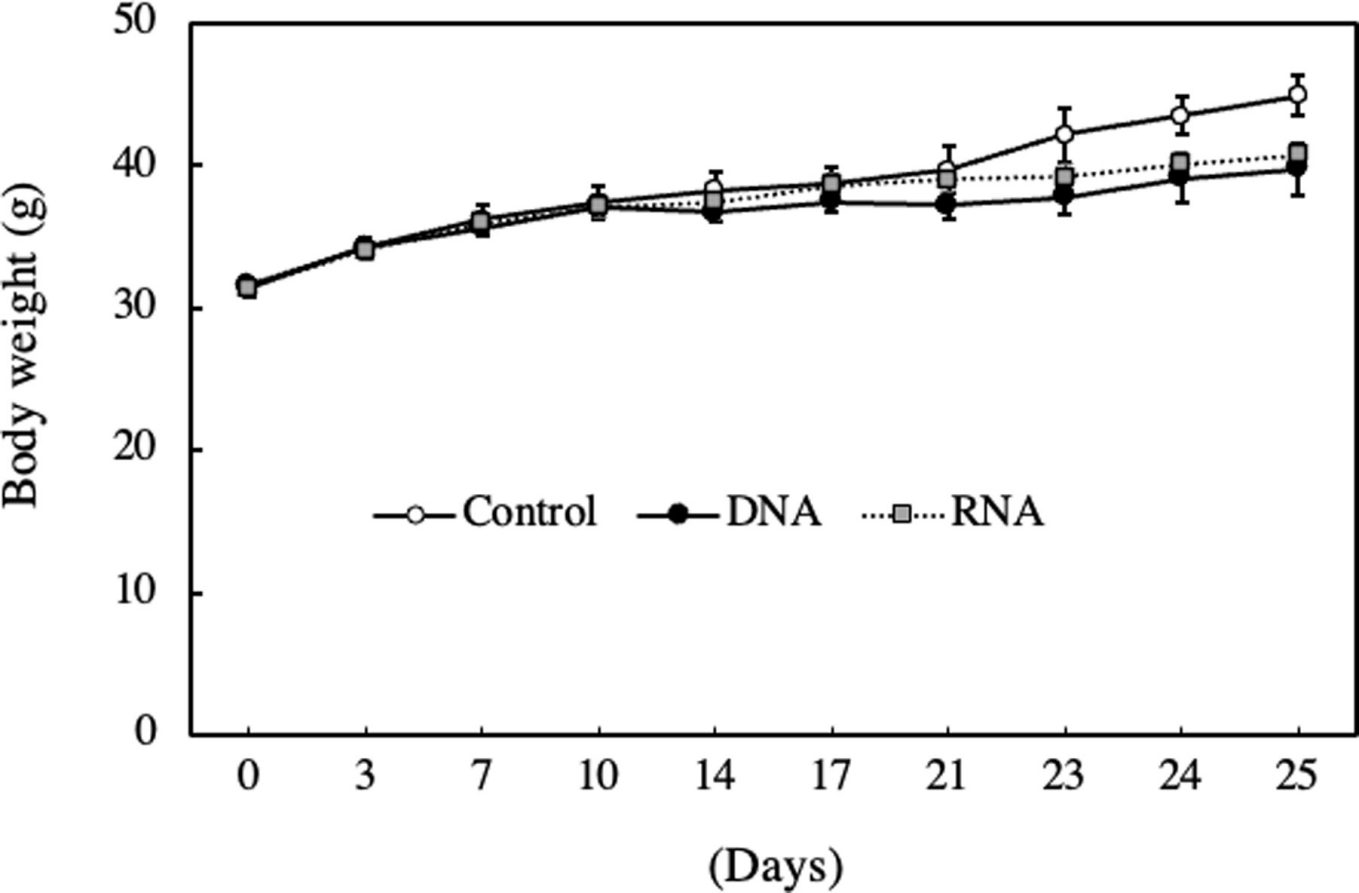
Effect of DNA and RNA on body weight in EAT cell-bearing mice. Mice were orally administered DNA or RNA (0.05 mg/g body weight) every 2 days for 10 days and then intraperitoneally administered 500 μl of EAT cell suspension adjusted to 1.0 x 10^5^ cells/ml with PBS. After the administration of EAT cells, DNA or RNA was orally administered every day. Data are presented as mean ± SE (n = 5–6).

**Fig 3:**
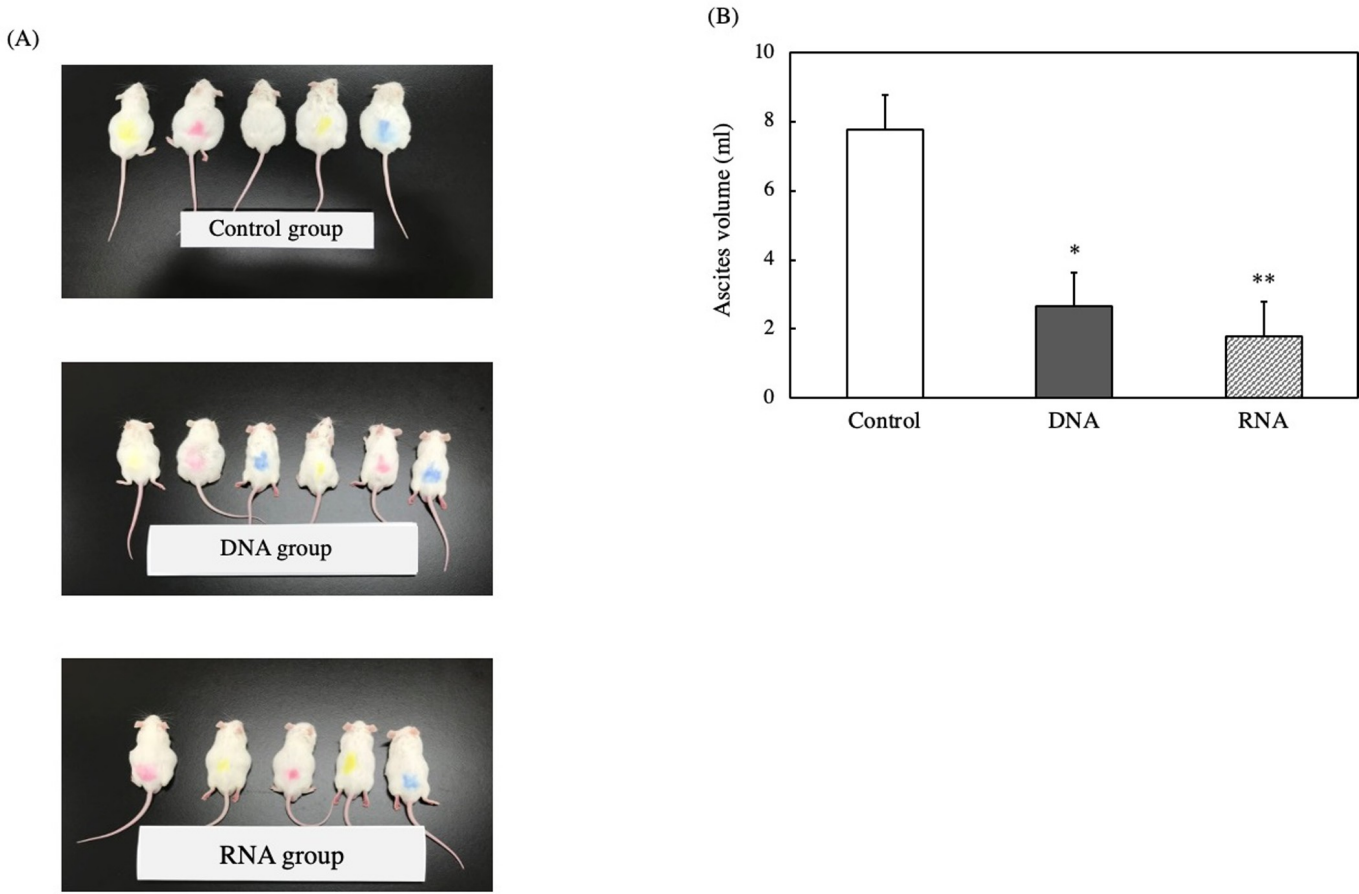
Effect of DNA and RNA on the volume of ascites fluid in EAT cell-bearing mice. (A) shows the macroscopic figure. The colors attached to the mice were intended for distinguishing between mice within the housing cage, and not for distinguishing between groups. (B) presents the volume of ascites. The mice were orally administered 200 μl of ultrapure water containing 0.05 mg DNA or RNA per gram of body weight as samples. Ten days after the experiment started, 500 μl of EAT cell suspension adjusted to 1.0×10^5^ cells/ml with PBS was intraperitoneally administered to the mice, with the samples continuing to be administered orally on a daily basis. Twenty-five days after the start of the experiment, the mice were dissected under deep isoflurane inhalation anesthesia to minimize pain, and the volume of ascites was measured. The data are shown as mean ± SE (n = 5–6) and are considered significantly different relative to the control (**p < 0.01 and *p < 0.05).

The antitumor activity of nucleic acids in mice bearing EAT cells was investigated. The changes in body weight during experiment were shown in Fig 2, while macroscopic figures and ascites volumes on the final day of experiment were presented in Fig 3. From 10 days after intraperitoneal inoculation of EAT cells, the control group tended to gain more body weight than the groups administered with DNA or RNA. The macroscopic figures in Fig 3A suggested that the increase in the weight is likely due to increased volume of ascites. Furthermore, the volume of ascites in the groups administered with DNA or RNA was smaller than that in the control group (Fig 3B).

### Anti-tumor activity of nucleic acids in culture of EAT cells

The antitumor activity of nucleic acids was examined in an EAT cell culture system to further investigate their mechanism of action, following the discovery of their anticancer properties in mice bearing EAT cells.

**Fig 4:**
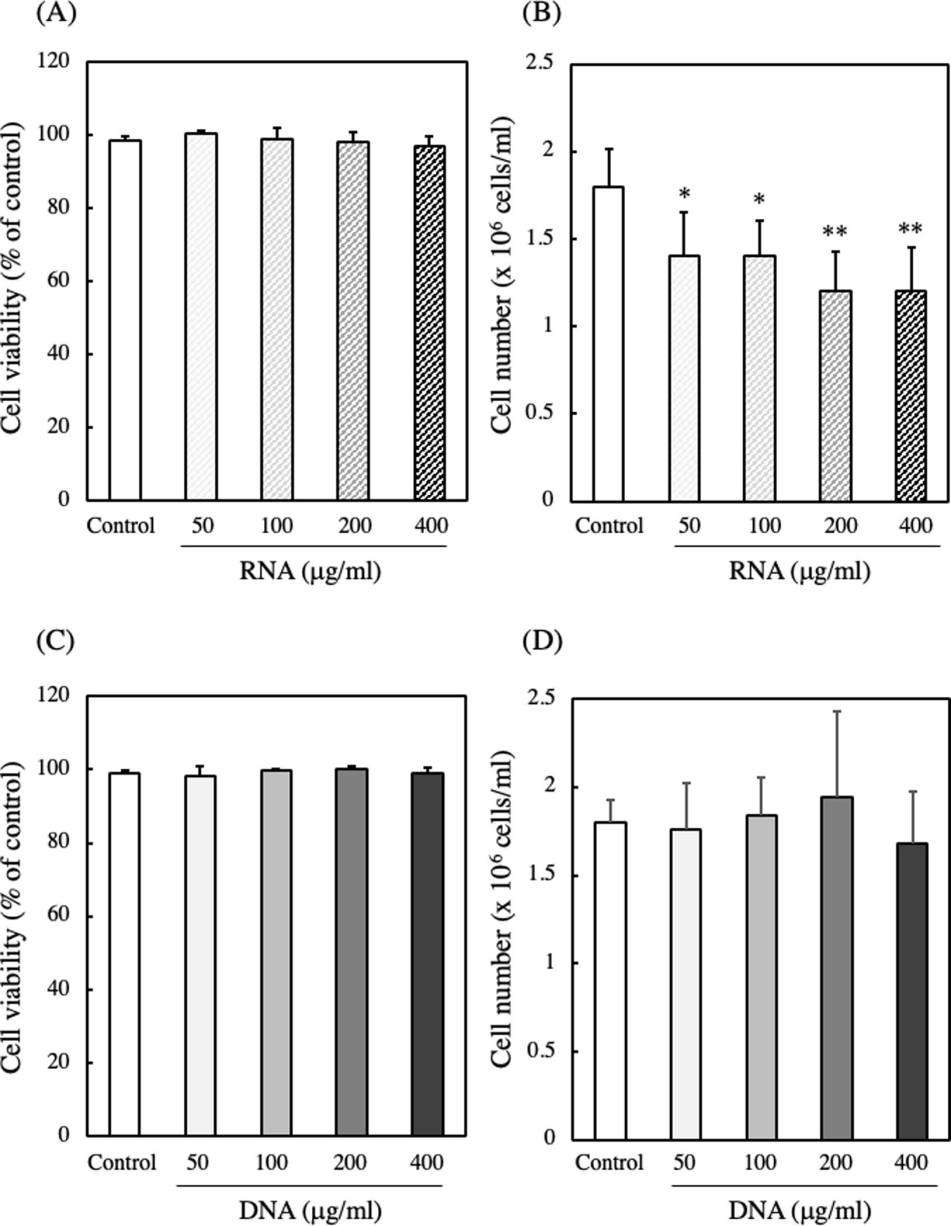
Effect of RNA and DNA on the viability and number of EAT cells. (A and B) EAT cells were cultured with various concentrations of RNA (0−400 μg/ml) for 24 h. (C and D) EAT cells were cultured with various concentrations of DNA (0−400 μg/ml) for 24 h. The viability (A and C) and number (B and D) of EAT cells were assessed using a trypan blue assay. Data are presented as mean ± SD (n = 5). The data were found to be significantly different from the control (**p < 0.01 and *p < 0.05).

When EAT cells were cultured with RNA and DNA at concentrations up to 400 μg/ml for 24 hours, the addition of RNA or DNA did not reduce cell viability. These results indicate that RNA or DNA was not cytotoxic against EAT cells at concentrations up to 400 μg/ml. However, the viable number of EAT cells decreased in a dose-dependent manner with the addition of RNA, while the addition of DNA did not affect the cell count (Fig 4A–4D).

These results suggest that the decrease in viable number of EAT cells with the addition of RNA occurred not due to cell death but rather due to the cessation of cell proliferation.

### Effect of DNA hydrolysate on cell viability and cell number of EAT cells

**Fig 5:**
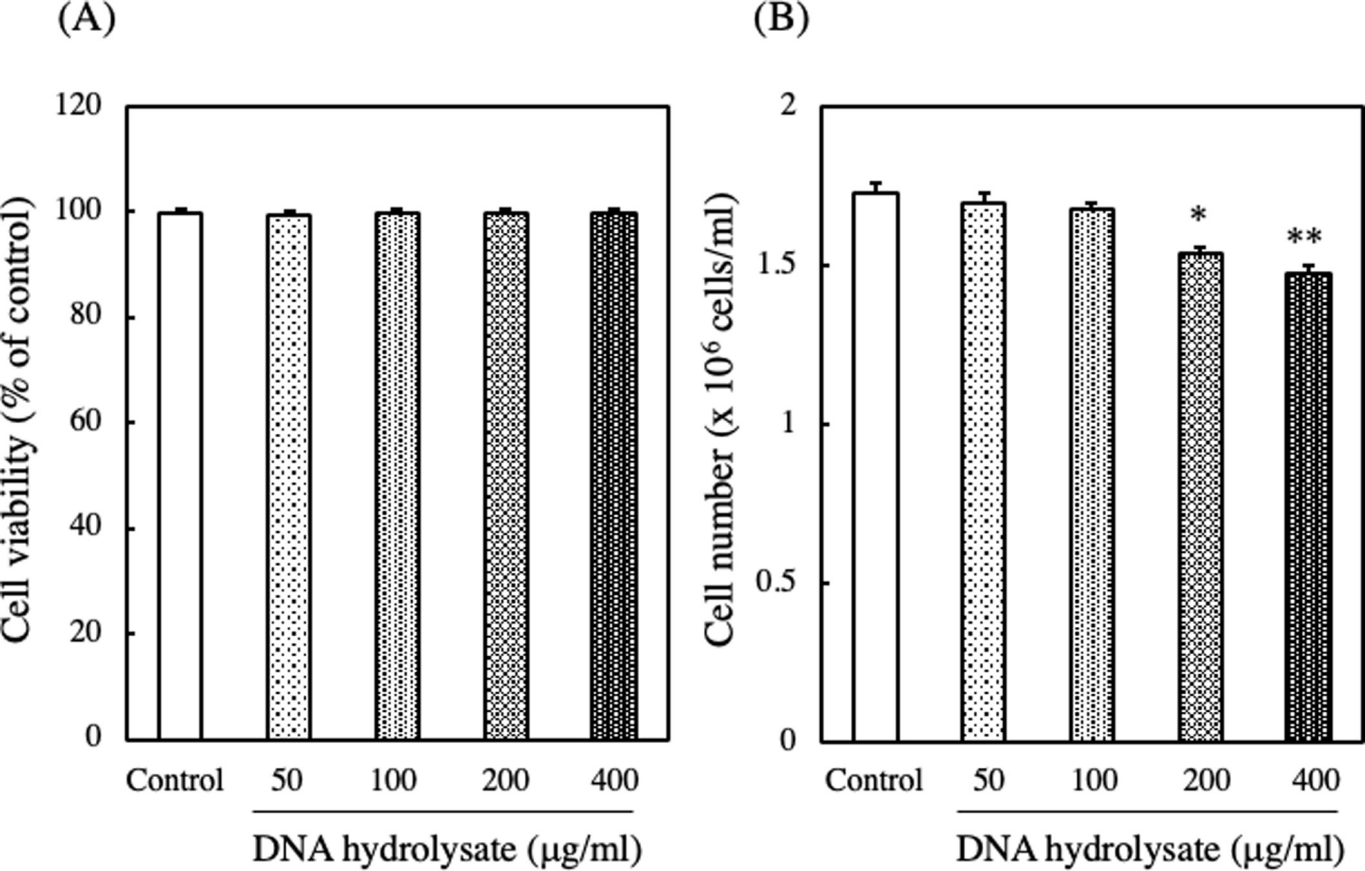
Effect of DNA hydrolysate on the viability and number of EAT cells. EAT cells were cultured with various concentrations of DNA hydrolysate (0−400 μg/ml) for 24 h. The viability (A) and number (B) of EAT cells were assessed using a trypan blue assay. Data are presented as mean ± SD (n = 5). The data were found to be significantly different from the control (**p < 0.01 and *p < 0.05).

In the animal experiments, the intake of DNA suppressed ascites cancer, but when DNA was added to the cultured EAT cells, no effects were observed. This suggested that for DNA to exhibit its effects, digestion might be necessary. Subsequently, we investigated the impact of hydrolyzed DNA on EAT cells. When EAT cells were cultured with 50–400 μg/ml of DNA hydrolysate for 24 hours, the addition of DNA hydrolysate did not decrease their cell viabilities. However, the cell numbers were significantly reduced with the addition of DNA hydrolysate at 200 and 400 μg/ml (Fig 5A and 5B).

**Fig 6:**
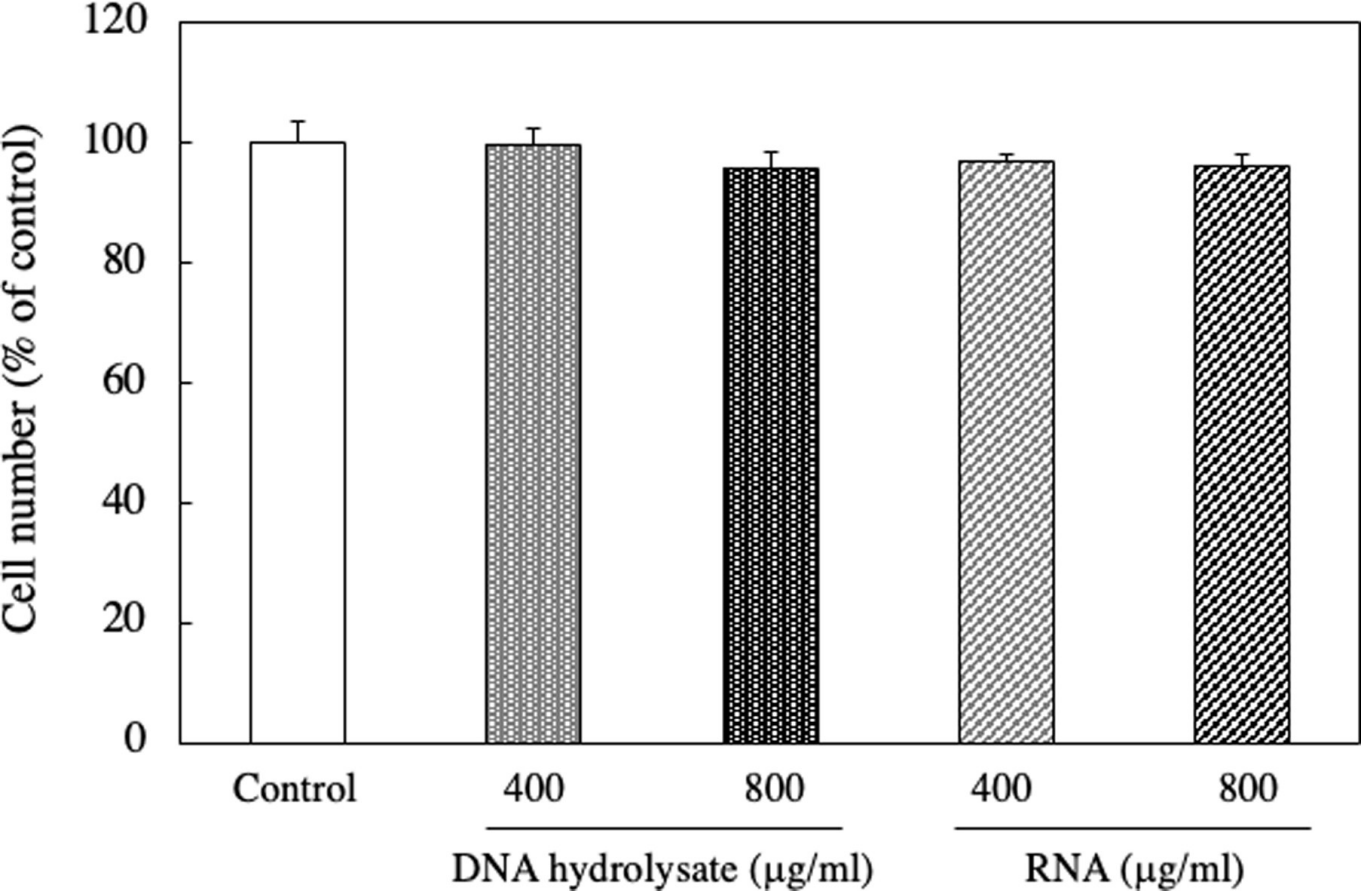
Effect of RNA and DNA hydrolysate on the cell number of 3T3-L1 cells. 3T3-L1 cells were cultured with 400 and 800 μg/ml of RNA or DNA hydrolysate for 24 h. The cell number was assessed using a neutral red assay. Data are presented as mean ± SD (n = 5).

To determine whether the reduction of EAT cell number by adding DNA hydrolysate and RNA was specifically effective on cancer cells, we investigated the effects of DNA hydrolysate and RNA addition on the cell proliferation of 3T3-L1, a type of normal cells. As a result, no significant impact on the cell count of 3T3-L1 cells was observed at concentrations of 400 and 800 μg/ml (Fig 6). This result suggests that the actions of DNA hydrolysate and RNA were specific to cancer cells.

### Effect of nucleoside transporter inhibitor on RNA- or DNA hydrolysate-treated EAT cells

**Fig 7:**
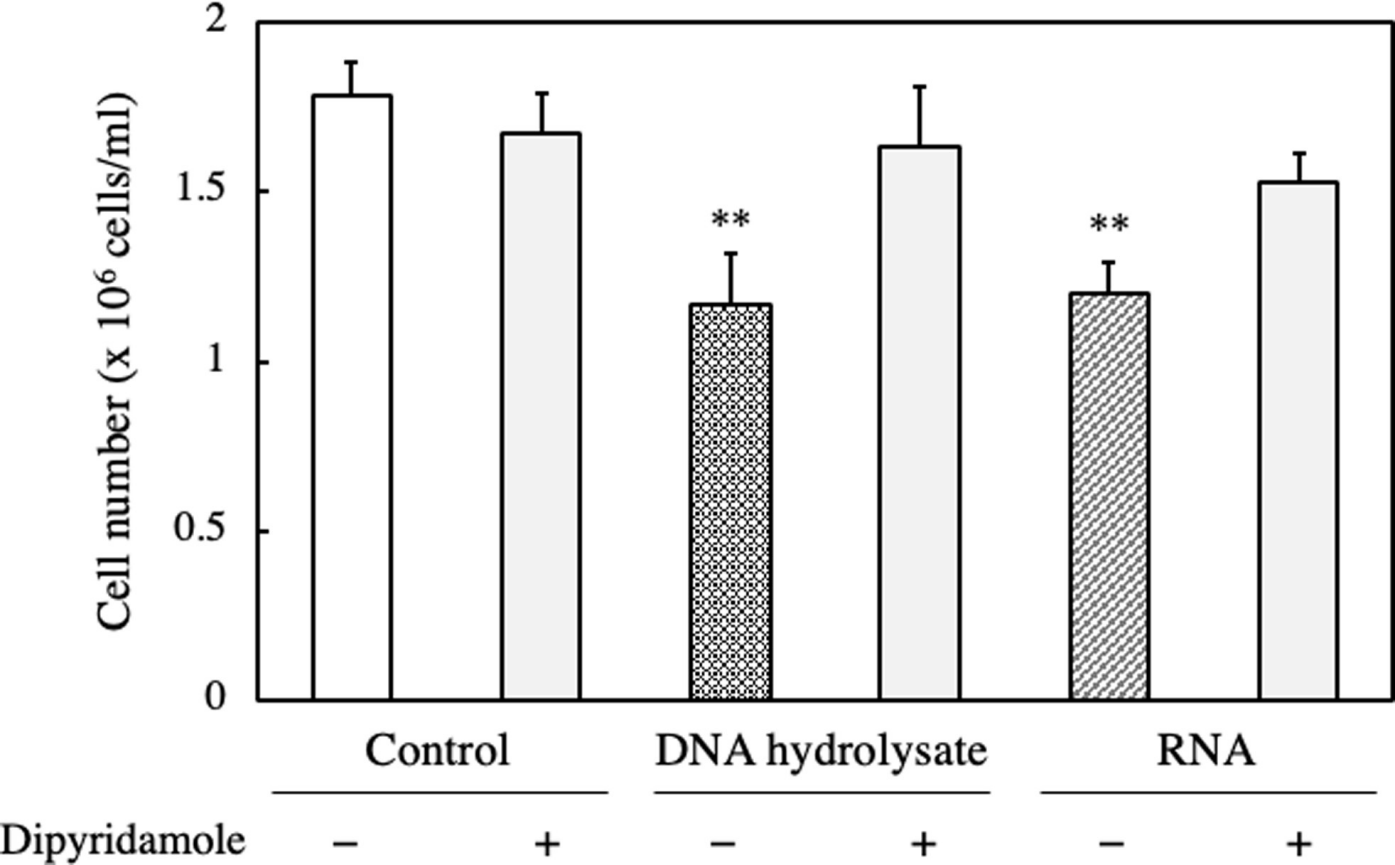
Effect of nucleoside transporter inhibitor on the cell number of RNA and DNA hydrolysate-treated EAT cells. EAT cells were cultured with 200 μg/ml of DNA hydrolysate or RNA and with or without 10 μM of dipyridamole, an inhibitor of nucleoside transporter, for 24 h. The cell number was assessed using a trypan blue assay. Data are presented as mean ± SD (n = 5). The data were found to be significantly different from the control (**p < 0.01).

The anti-proliferative effect of RNA- or DNA hydrolysate treatment suggests that oligonucleotides, nucleotides, or nucleosides were effective in inhibiting cell proliferation mediated by nucleic acids. Nucleosides play important roles in various cellular processes. Therefore, to confirm whether the effects of RNA or DNA hydrolysates were mediated by the nucleotides contained within them, we conducted verification using inhibitors of nucleoside transporters and examined the effect of dipyridamole, an inhibitor of nucleoside transporters, on the anti-proliferative activity of RNA- or DNA hydrolysates. The activities of RNA- or DNA hydrolysates were suppressed with the treatment of the nucleoside transporter inhibitor, indicating that nucleosides possess an anti-proliferative effect (Fig 7).

### Effect of various nucleosides on cell viability and cell number of EAT cells

**Fig 8:**
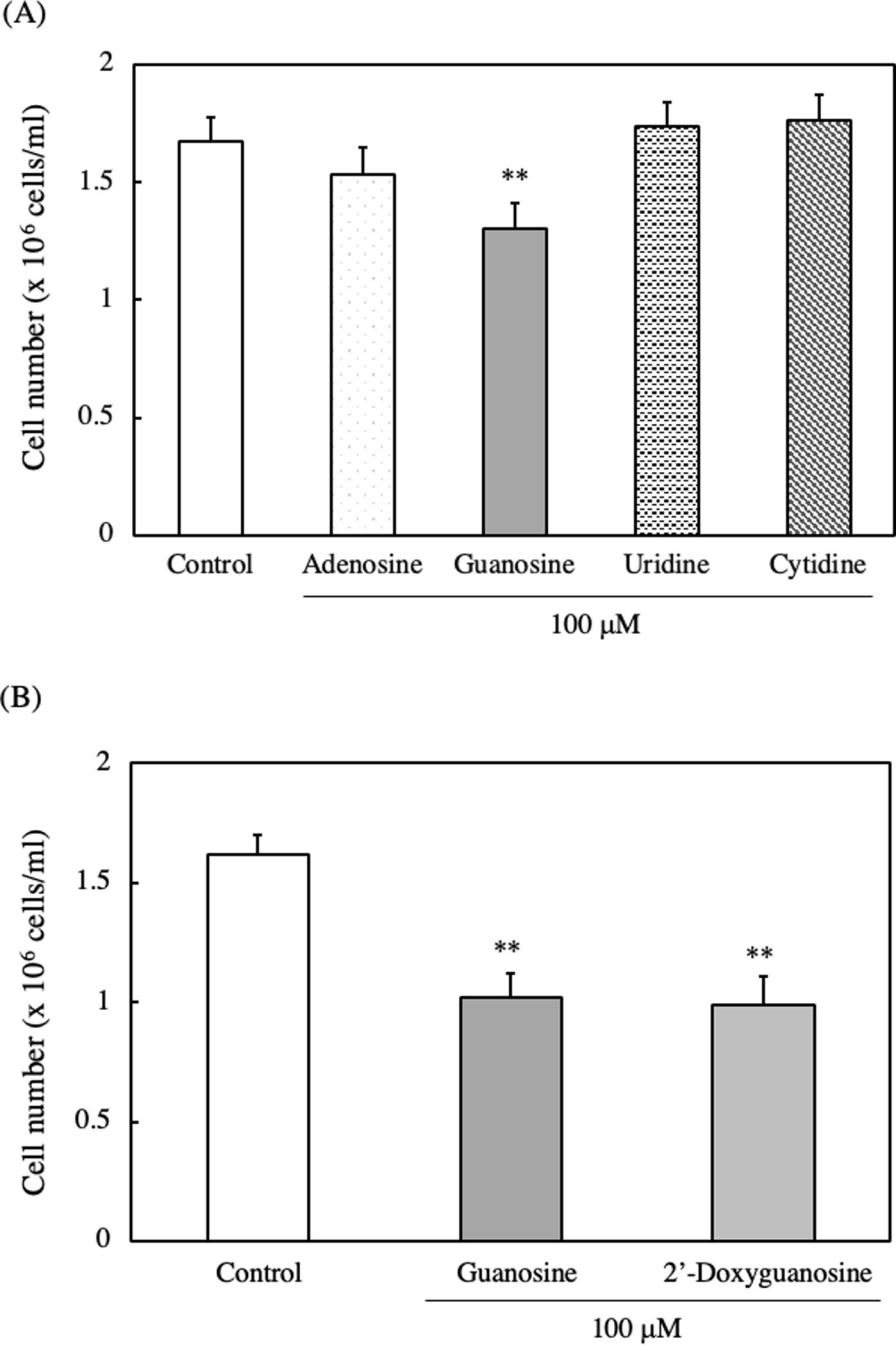
Effect of various nucleosides on the cell number of EAT cells. EAT cells were cultured with (A) 100 μM of adenosine, guanosine, uridine, or cytidine, and (B) 100 μM guanosine or 2-deoxyguanosine for 24 h. The cell number was assessed using a trypan blue assay. Data are presented as mean ± SD (n = 6). The data were found to be significantly different from the control (**p < 0.01).

We conducted an investigation to determine which nucleosides exhibit anti-proliferative effects. EAT cells were cultured with different nucleosides, including adenosine, guanosine, uridine or cytidine, for 24 h. Among them, only guanosine significantly decreased the cell number of EAT cells (Fig 8A). Since we previously observed a decrease in cell numbers with the addition of DNA hydrolysate, we further examined the effect of 2’-deoxyguanosine on the cell number of EAT cells. Interestingly, the treatment of 2’-deoxyguanosine resulted in a decrease in cell number comparable to that observed in guanosine-treated EAT cells (Fig 8B).

### Effect of inhibitors of nucleoside transporter on the anti-proliferation activity of nucleic acids-treated EAT cells

**Fig 9:**
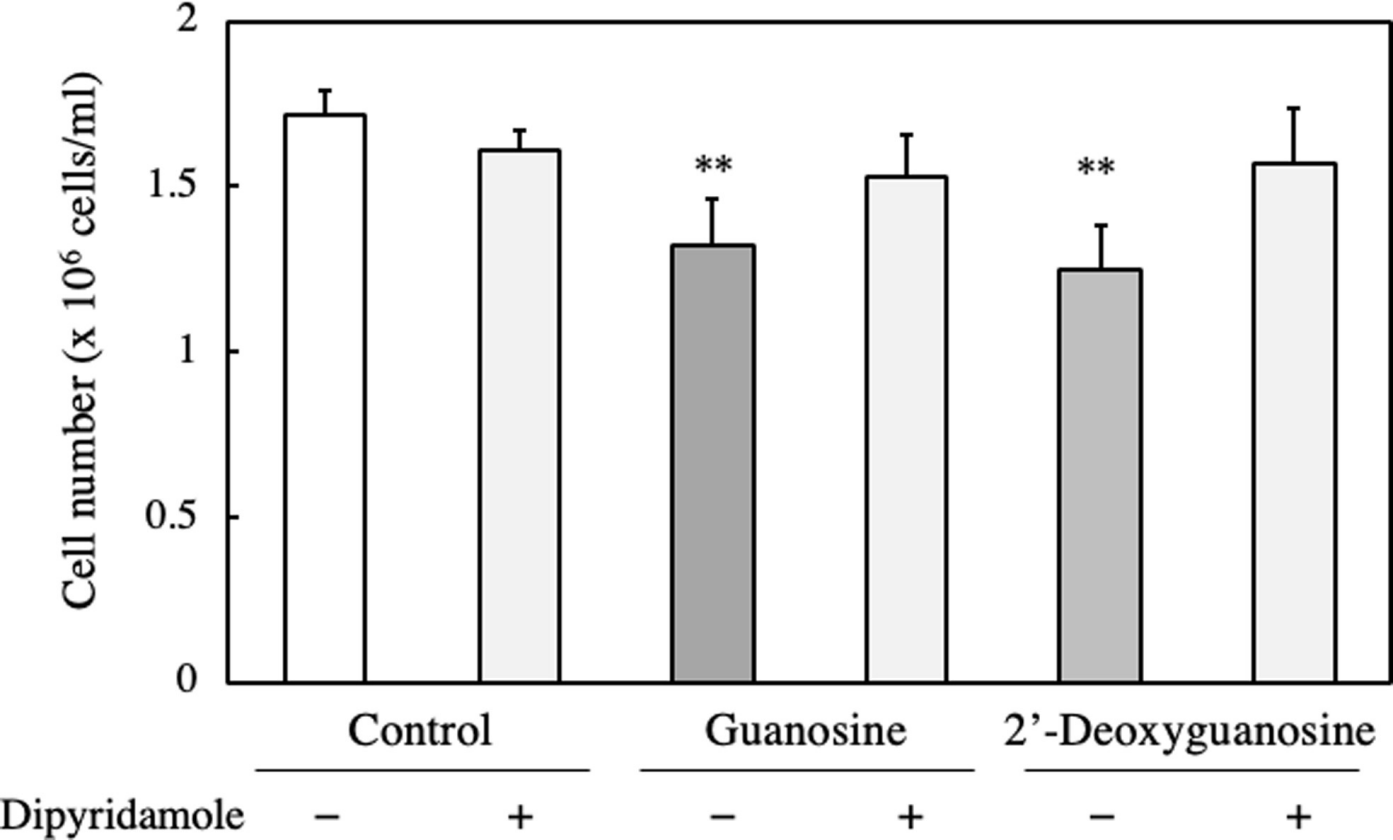
Effect of nucleoside transporter inhibitor on the cell number of RNA and DNA hydrolysate-treated EAT cells. EAT cells were cultured with 100 μM guanosine or 2’-deoxyguanosine, with or without 10 μM of dipyridamole, an inhibitor of nucleoside transporter, for 24 h. The cell number was assessed using a trypan blue assay. Data are presented as mean ± SD (n = 6). The data were found to be significantly different from the control (**p < 0.01).

**Fig 10:**
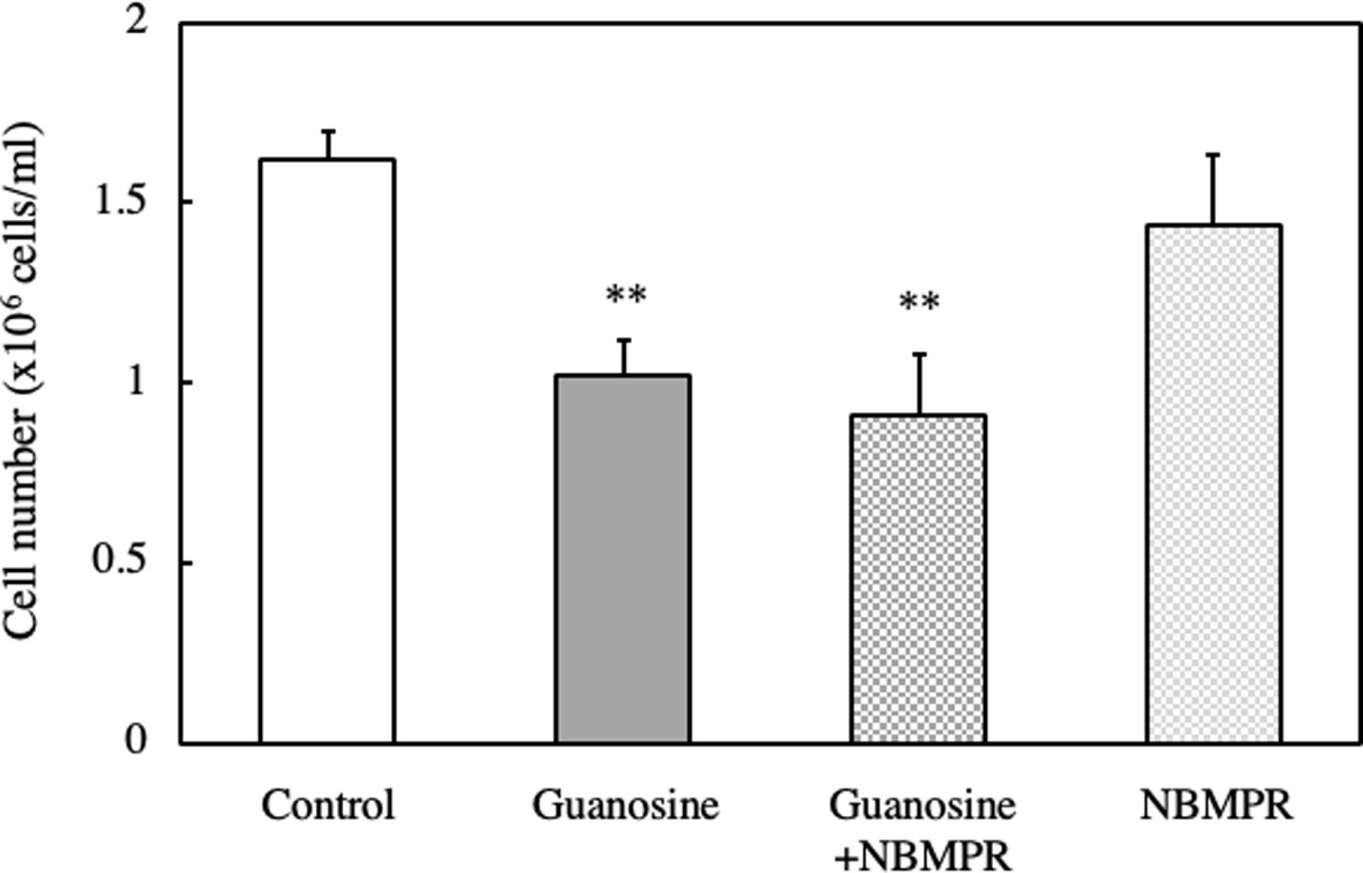
Effect of NBMPR, a nucleoside transporter inhibitor, on the cell number of RNA and DNA hydrolysate-treated EAT cells. EAT cells were cultured with 100 μM guanosine with or without 0.5 μM of NBMPR, an inhibitor of nucleoside transporter, for 24 h. The cell number was assessed using a trypan blue assay. Data are presented as mean ± SD (n = 5).

Mammals have two types of nucleoside transporters: sodium-independent equilibrative nucleoside transporters (ENTs) and sodium-dependent concentrated nucleoside transporters (CNTs). Among the ENTs, there are four subtypes known as ENT1, ENT2, ENT3 and ENT4, which are present in various tissues and facilitate the transport of nucleosides across cell membrane. For this study, we used two inhibitors of nucleoside transporter: dipyridamole [20] and nitrobenzylthioinosine (NBMPR) [21]. Dipyridamole is a non-selective inhibitor for ENT1 and ENT2 while NBMPR has low sensitivity for ENT2 [22]. It is worth noting that both inhibitors have low sensitivity for CNTs. In our experiment, we investigated the effect of dipyridamole and NBMPR on anti-proliferation activity of guanosine and 2’-deoxyguanosine. Interestingly, we found that the treatment of dipyridamole suppressed the anti-proliferation activity with both nucleosides (Fig 9). On the other hand, NBMPR did not show any suppression of the anti-proliferation (Fig 10).

### Effect of guanosine on DNA contents in EAT cells

**Fig 11:**
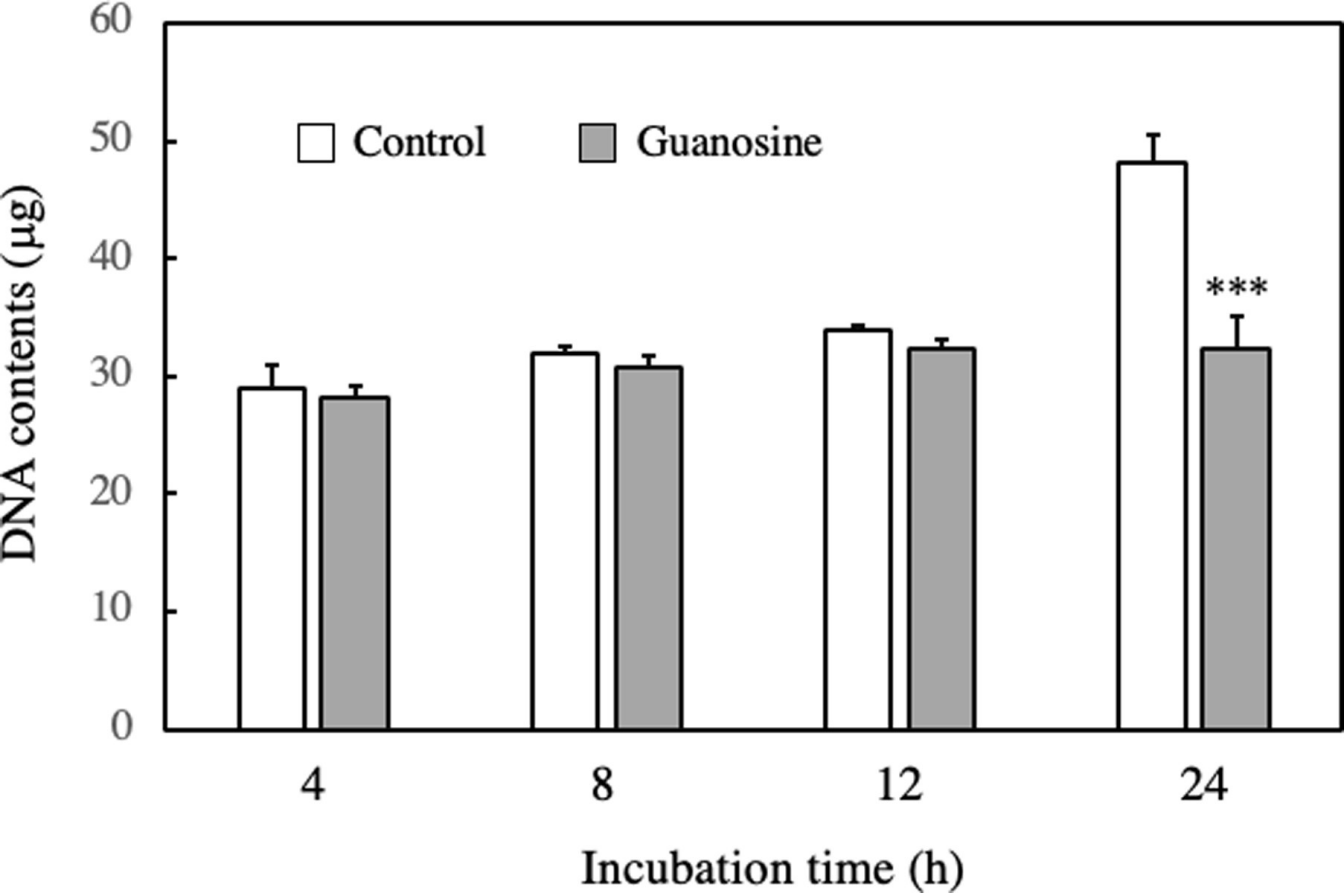
Effect of guanosine on DNA content of EAT cells. EAT cells were cultured with 100 μM guanosine for 4–24 h. The DNA contents of EAT cells were measured using the Burton procedure. Data are presented as mean ± SD (n = 6). The data were found to be significantly different from the control (***p < 0.001).

To investigate the stage at which the proliferation of EAT cells is inhibited by guanosine or 2’-deoxyguanosine, we quantified the DNA content of EAT cells at various incubation times. We examined the intracellular DNA contents in EAT cells cultured with or without 100 μM guanosine for 4, 8, 12, or 24 h (Fig 11). No significant changes in intracellular DNA content were observed until 12 h of culture between control cells and guanosine-treated cells. However, at 24 h, the DNA content in the control cells significantly increased, while the guanosine-treated cells did not exhibit an increase in DNA content. These results indicate that guanosine treatment inhibited DNA synthesis in EAT cells.

### Effect of guanosine on DNA synthesis in EAT cells

**Fig 12:**
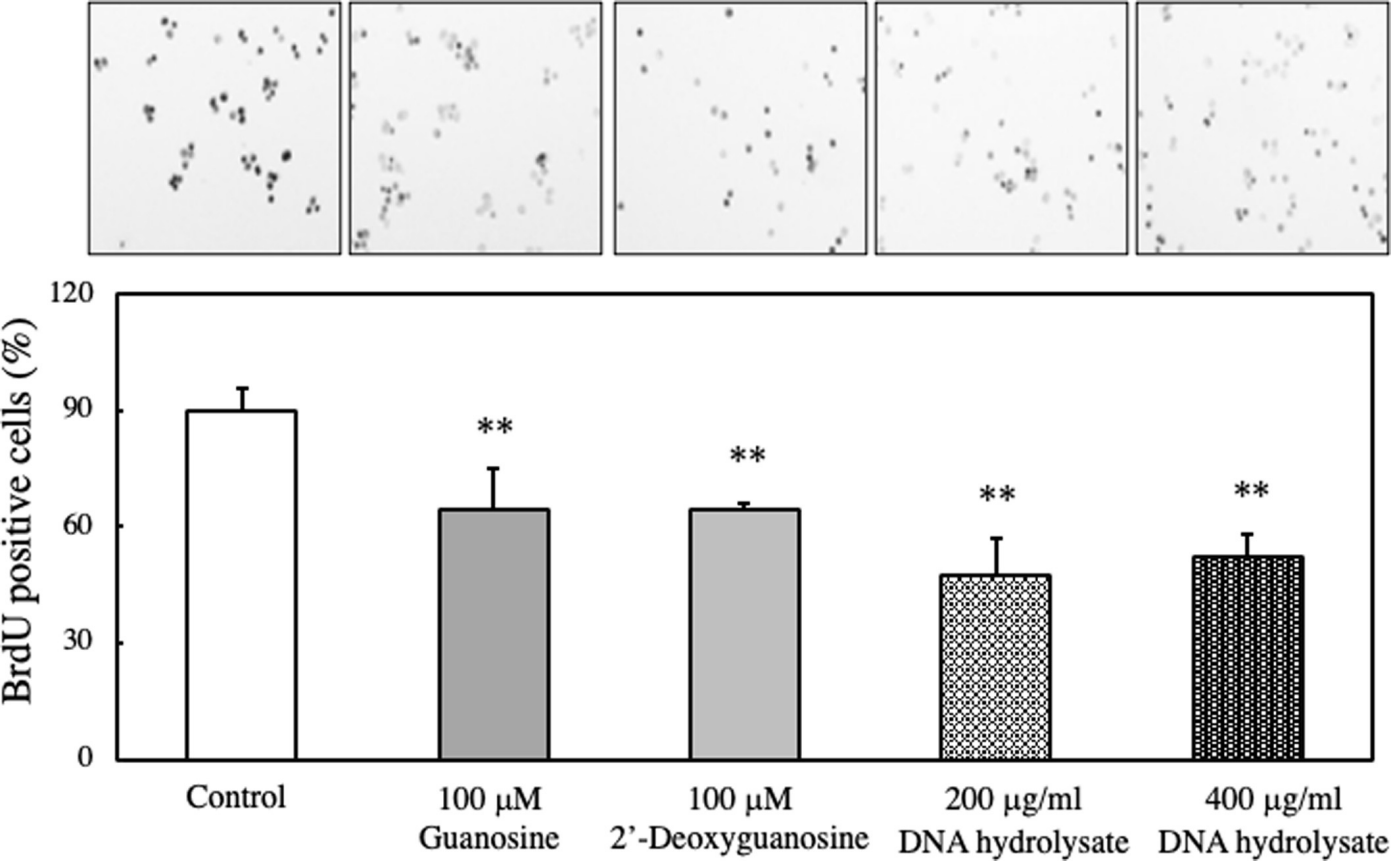
Effect of guanosine, 2-deoxyguanosine, or DNA hydrolysate on DNA synthesis of EAT cells. EAT cells were cultured with BrdU (100 μM) and guanosine, 2-deoxyguanosine, or DNA hydrolysate for 24 h. DNA synthesis was assessed using the BrdU assay, and immunostaining images show BrdU-positive cells. The percentage of BrdU-positive cells was calculated by dividing the BrdU-positive cell number by the total cell number. Data are presented as mean ± SD (n = 5). The data were found to be significantly different from the control (**p < 0.01).

To further confirm the inhibition of DNA synthesis by guanosine, we employed the BrdU method. As shown in Fig 12, the addition of guanosine, 2’-deoxyguanosine, or DNA hydrolysate significantly reduced the number of BrdU-positive cells, indicating a cell’s arrest in the G1 phase.

**Fig 13:**
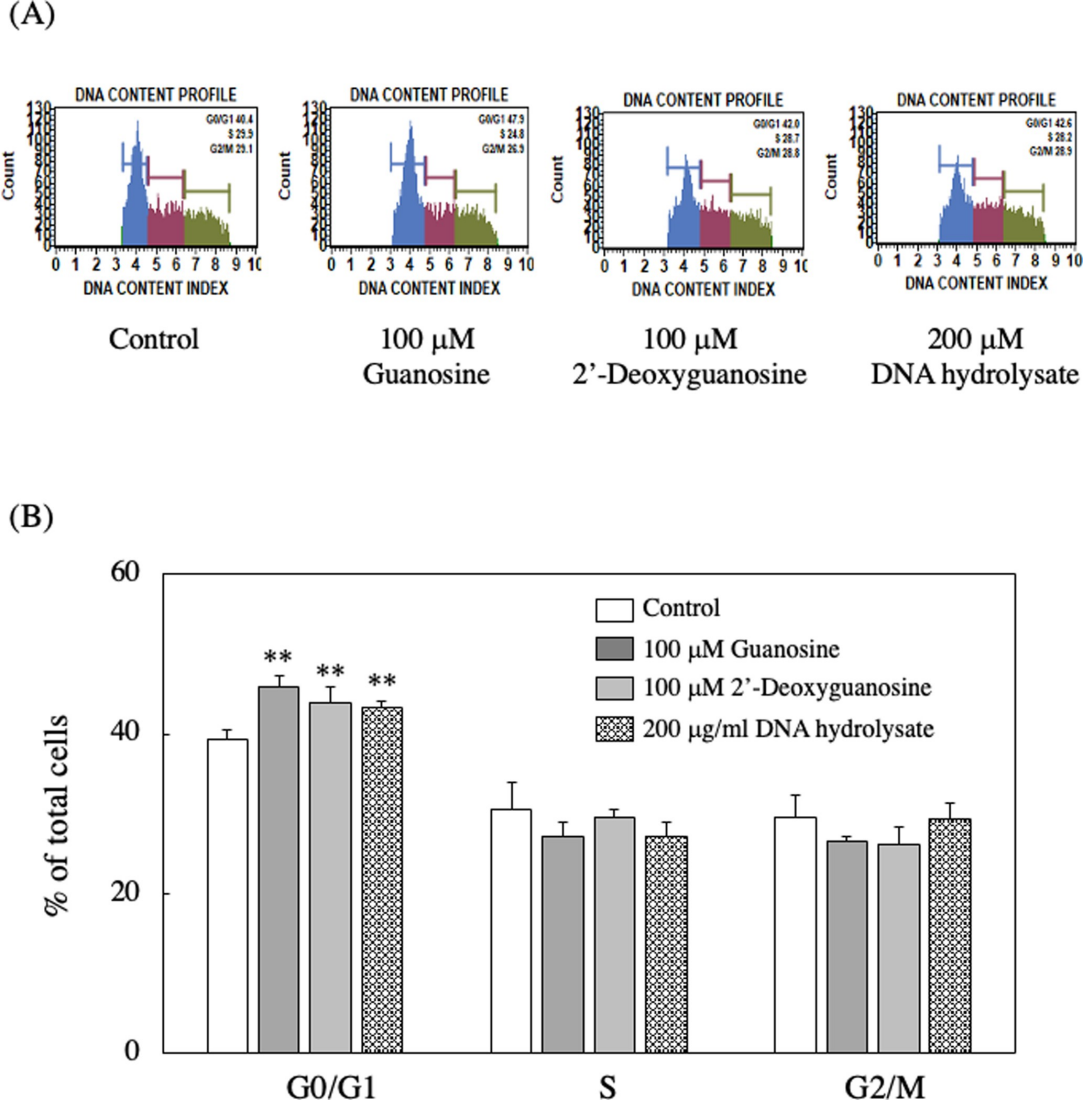
Effect of guanosine, 2-deoxyguanosine, or DNA hydrolysate on cell cycle progression of EAT cells. EAT cells were cultured with guanosine, 2-deoxyguanosine, or DNA hydrolysate for 24 h. Cell cycle was analyzed using the Muse^Ⓡ^ Cell Analyzer. (A) Histograms of DNA content show the distribution of cell cycle phases (G0/G1, S, and G2/M) of EAT cells. (B) The ratio of the cell cycle stages of EAT cells was analyzed. Data are presented as mean ± SD (n = 4). The data were found to be significantly different from the control (**p < 0.01).

Additionally, to clarify this result in more detail, the cell cycle progression was analyzed. As shown in Fig 13, the number of G0/G1 phase cells was significantly increased by incubating EAT cells with guanosine, 2’-deoxyguanosine, or DNA hydrolysate. These results suggested that guanosine, 2’-deoxyguanosine, or DNA hydrolysate was induced G1phase arrest of the cell cycle.

**Fig 14:**
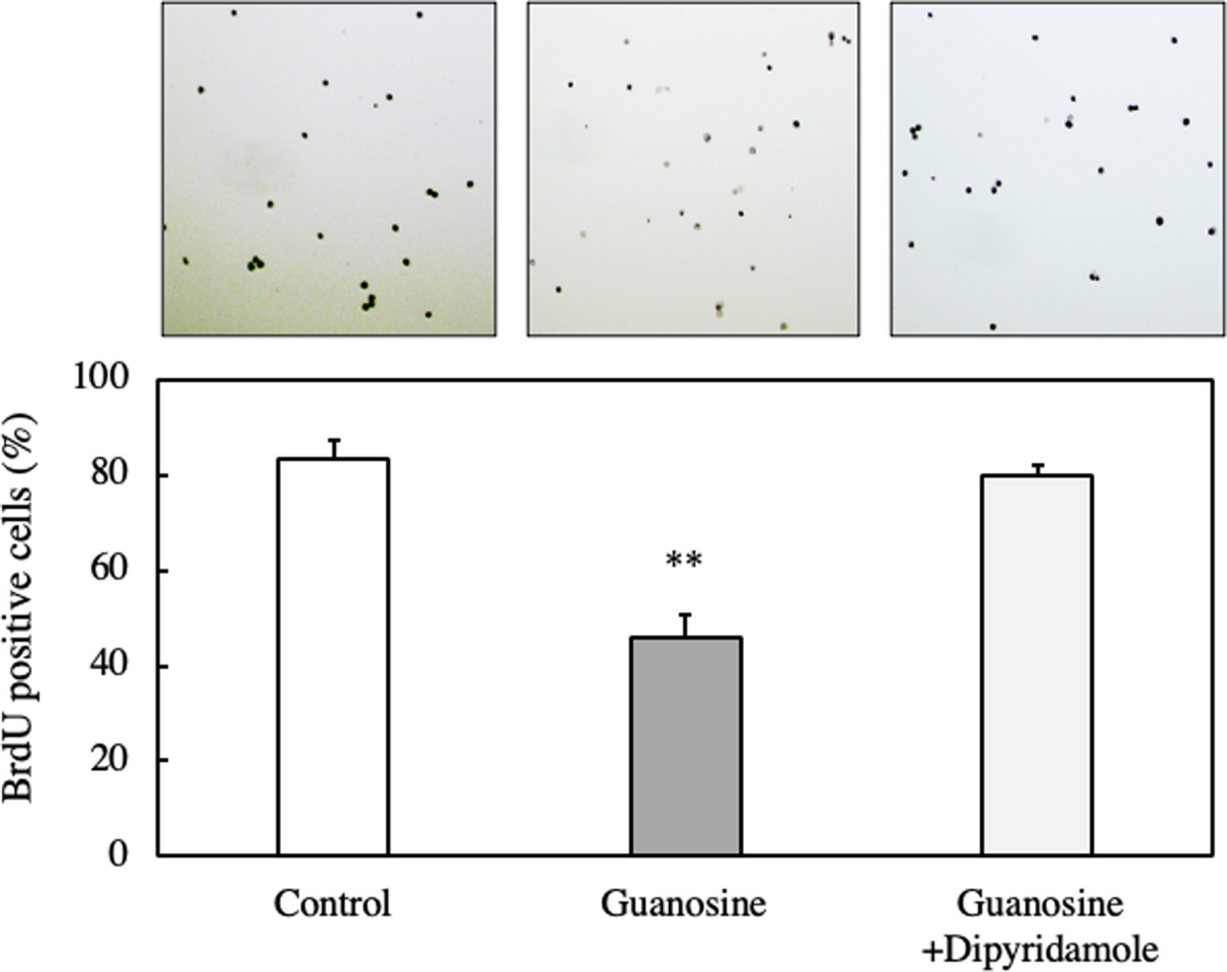
Effect of guanosine and dipyridamole, a nucleoside transporter inhibitor, on DNA synthesis of EAT cells. EATC were cultured with BrdU (100 μM) and dipyridamole (10 μM) for 24 h. DNA synthesis was assessed using the BrdU assay. Immunostaining images show BrdU-positive cells. The percentage of BrdU-positive cells was calculated by BrdU-positive cell number relative to the total cell number. Data are presented as mean ± SD (n = 5). The data were considered significantly different relative to the control (**p < 0.01).

On the other hand, the suppression of BrdU-positive cells by guanosine was reversed upon the addition of dipyridamole, a nucleoside transporter inhibitor (Fig 14).

### Effect of guanosine on the expression of C/EBPβ gene in EAT cells

**Fig 15:**
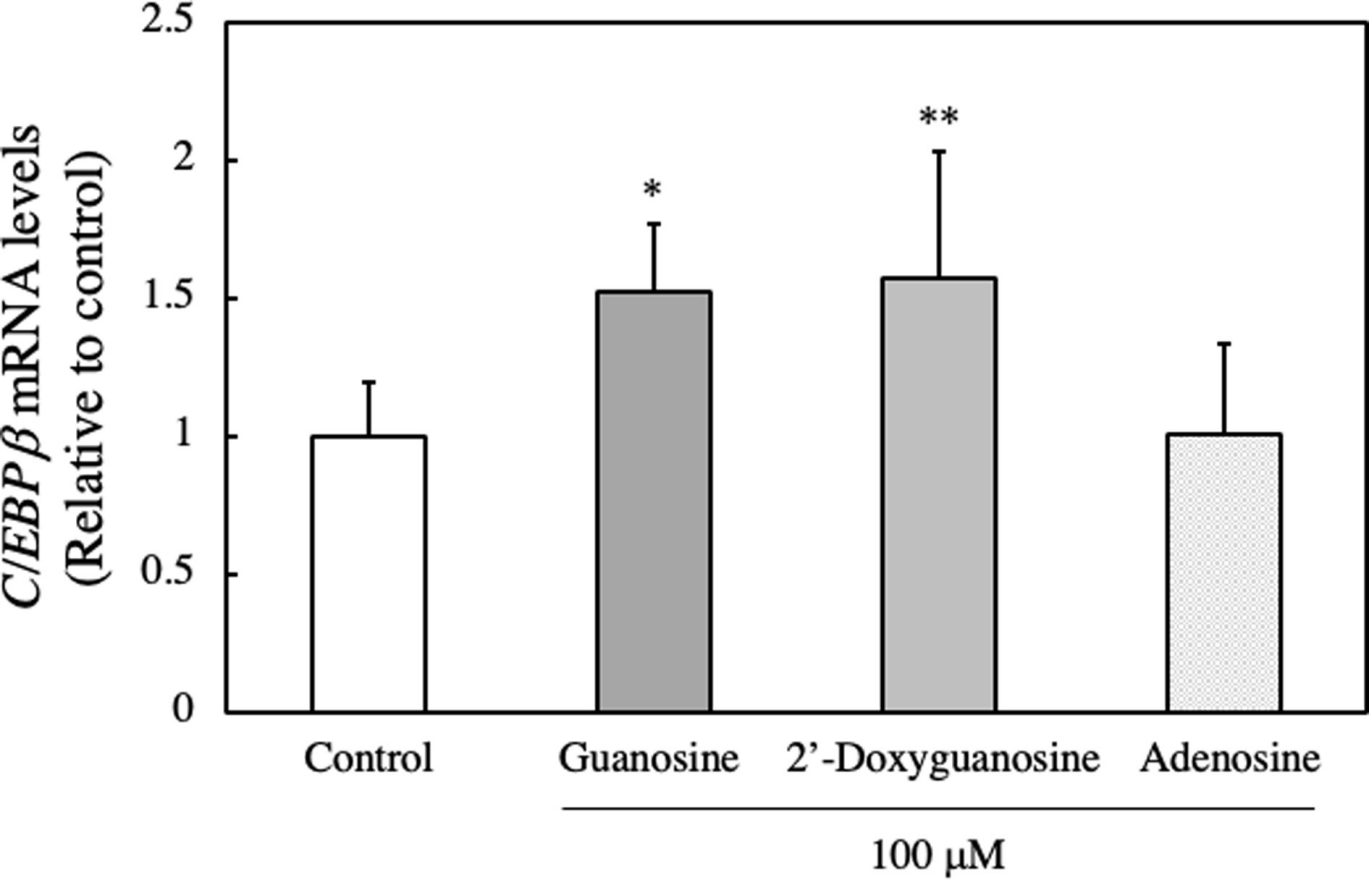
Effect of guanosine, 2’-deoxyguanosine or adenosine on the gene expression of C/EBPβ. mRNA expression was analyzed by qRT-PCR 24 h after incubation of guanosine-, 2’-deoxyguanosine- or adenosine-treated EAT cells. Data are presented as mean ± SD (n = 3).

**Fig 16:**
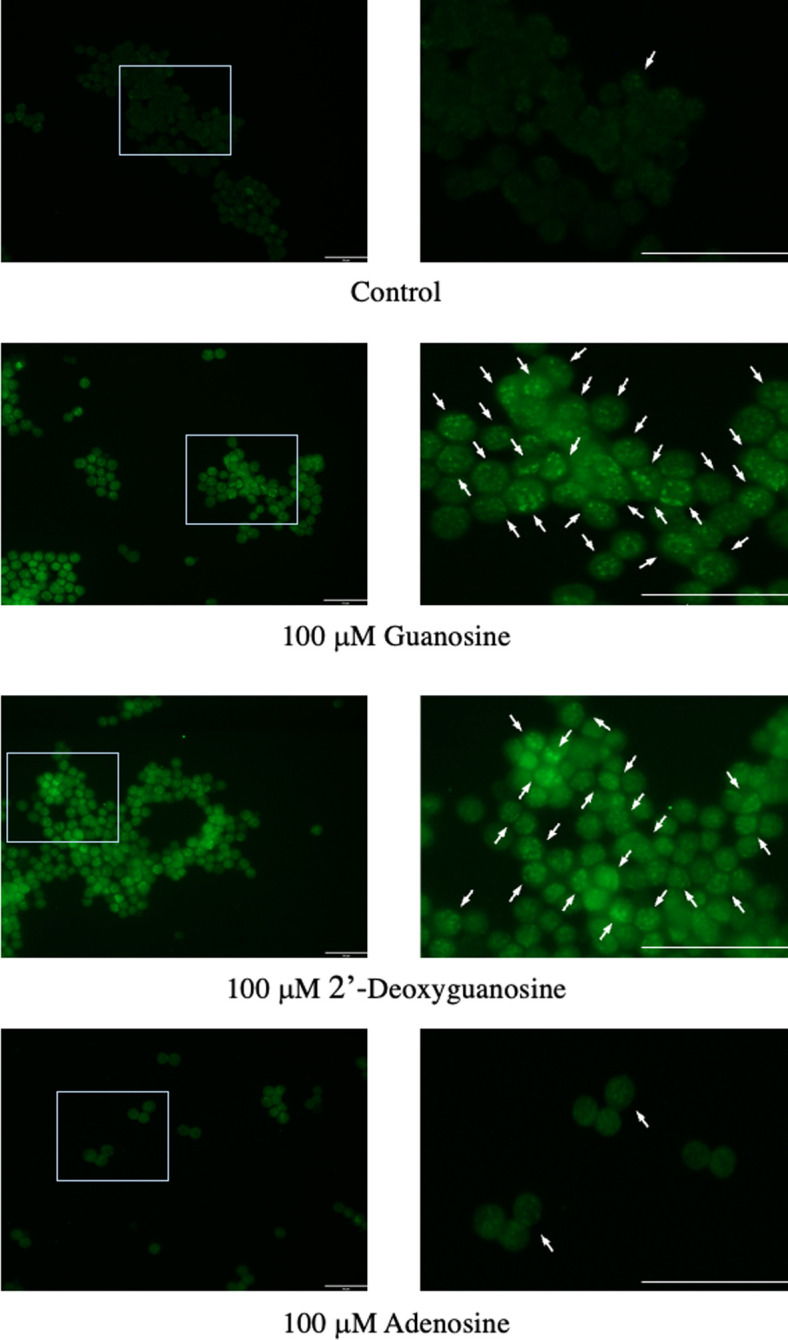
The intracellular localization of C/EBPβ in guanosine-, 2’-deoxyguanosine- or adenosine-treated EAT cells. EAT cells were cultured for 16 h after the addition of these nucleosides, fixed and subjected to *in site* immunofluorescence analysis. The photo on the right enlarges the area enclosed by the frame on the left. Arrows indicated the binding of C/EBPβ to satellite DNA. Bar = 50 μm.

C/EBPβ plays critical roles in the regulation of cellular proliferation and differentiation, apoptosis, metabolism, transformation, and inflammation [23, 24]. It has been documented that C/EBPβ shares numerous biological properties with C/EBPα, including the inhibition of proliferation and tumorigenesis, as well as the promotion of differentiation. Additionally, Buck *et al*. reported that elevated C/EBPβ expression in HepG2 hepatoma cells led to cell cycle arrest in the G1 phase [25]. In this study, we investigated the impact of guanosine, 2’-deoxyguanosine, and adenosine on the gene expression of C/EBPβ. As depicted in Fig 15, the gene expression levels of C/EBPβ in guanosine or 2’-deoxyguanosine-treated EAT cells increased by 1.527 or 1.570-fold, respectively, compared with control cells. However, the gene expression level of C/EBPβ in adenosine-treated EAT cells showed only a 1.006 fold change. The results suggest that the expression of C/EBPβ in EAT cells is upregulated by guanosine or 2’-deoxyguanosine. Subsequently, the intracellular localization of C/EBPβ was examined through *in site* immunofluorescence 16 h after the addition of guanosine, 2’-deoxyguanosine or adenosine. In the guanosine or 2’-deoxyguanosine addition group, the binding of C/EBPβ to satellite DNA were observed. However, in the control and adenosine addition groups, a lower binding of C/EBPβ was noted (Fig 16).

## Discussion

This study was conducted to explore the impact of dietary nucleic acids and nucleosides on cancer cells, utilizing Ehrlich ascites tumor (EAT) cells recognized for their rapid growth. Our investigation led to the groundbreaking discovery that guanosine and 2’-deoxyguanosine, absorbed through nucleoside transporters, effectively hinder the proliferation of EAT cells by impeding the transition from the G1 phase to the S phase. Remarkably, this inhibition is associated with an increase in the expression of C/EBPβ induced by guanosine and 2’-deoxyguanosine. Immunostaining techniques further revealed the diffusion of C/EBPβ into the nucleus, confirming its presence. Consequently, our findings suggest a novel mechanism wherein guanosine or 2-deoxyguanosine induces G1 arrest in cancer cells through the activation of C/EBPβ.

Dietary nucleic acids have been found to have various physiological effects *in vivo*, other than being recycled through the salvage pathway. Their effects have attracted attention, and the physiological effects of dietary nucleic acids reported so far include the maintenance of immune responses [1–3], suppression of inflammation in adipose tissue [4], improvement of glucose tolerance [4], and protection of liver function [5, 26]. In this study, we discovered that dietary nucleic acids and guanosine have an inhibitory effect on cancer cell proliferation. Interestingly, we observed that DNA exhibited anti-proliferative effects in the *in vivo* mouse model but not in the *in vitro* cell model. However, the anti-proliferative effect of DNA hydrolysate was evident in the *in vitro* cell model. These suggest that nucleotides and nucleosides produced through the digestion of nucleic acids act as active ingredients. Adenosine and guanosine have previously been identified as nucleosides with anticancer activity. Adenosine, when added externally to cells, induces apoptosis in various cancer cells through adenosine receptors (A1, A2a, A2b, or A3) and downstream signaling pathways [25–27]. Adenosine can also be taken up by nucleoside transporters and converted to AMP, leading to apoptosis through the activation of AMP-dependent protein kinase [28]. It is worth noting that adenosine-induced cytotoxicity occurs at relatively high concentrations, while lower concentrations of adenosine exhibit physiological effects without cytotoxicity [29]. As for guanosine, it has been reported to enhance the anticancer effects of chemotherapeutic agents such as acriflavine, 5’-deoxy5-fluorouridine, and temozolomide [30–33]. However, there have been no reports on the anticancer effects of guanosine alone. Our study reveals a novel finding that adenosine did not inhibit EAT cell proliferation, whereas guanosine alone suppressed the growth of cancer cells.

Excessive intake of purines such as guanine can lead to the overproduction of uric acid, resulting in hyperuricemia. However, recent research reports indicate that the increase in serum uric acid levels is primarily caused by the accumulation of visceral fat due to obesity [34] and the influence of alcohol intake [35], rather than the effects of purine intake. After being absorbed in the intestines, purines are metabolized into uric acid, with 400–600 mg/day excreted in the urine by the kidneys and 200–300 mg/day excreted in the intestines (extra-renal excretion). A decrease in uric acid excretion due to any abnormality in the body can lead to hyperuricemia. However, since 50–90% of purines ingested as part of the diet are excreted within 24 h in healthy individuals [36, 37] it is considered not to pose a health problem. Furthermore, reports indicate that strict purine intake restrictions result in only a slight decrease in serum uric acid levels [37]. Additionally, the influence on serum uric acid levels has been found to be significantly influenced by genetic factors. It has been revealed that 88.2% of patients who develop gout at the age of 20 or younger have mutations in the uric acid excretion transporter ABCG2 gene [38]. Moreover, a large-scale cohort meta-analysis study conducted in the United States in 2017 investigating the genetic polymorphisms related to healthy serum uric acid levels and dietary habits found that while the variation in serum uric acid levels due to dietary habits and dietary content was less than 1%, the serum uric acid levels of subjects with genetic mutations varied by 7.9%, strongly suggesting that genetic mutations are more strongly associated with increases in serum uric acid levels than dietary purine intake [39].

Nucleoside transporters play crucial roles in the physiological and pharmacological activities of nucleosides and their analogues. Mammals have five main nucleoside transporters, which can be classified into two classes: energy-requiring Na^+^-dependent concentrative nucleoside transporters (CNT) and Na^+^-independent equilibrative nucleoside transporters (ENTs) [40]. ENT1 and ENT2 are cell surface proteins involved in adenosine signaling, with ENT1 having higher substrate affinity. ENT3 acts as a lysosomal transporter, while ENT4 functions strongly under acidic conditions [41, 42]. In our study, we used 10 μM dipyridamole and 0.5 μM NBMPR as inhibitors of nucleoside transporters. Previous research by Ward et al. [22] reported the IC50 values of dipyridamole and NBMPR for human ENT1 and ENT2. The IC50 values of dipyridamole for ENT1 and ENT2 were 5.0 ± 0.9 nM and 356 ± 13 nM, respectively. On the other hand, the IC50 values of NBMPR for ENT1 and ENT2 are 0.4 ± 0.1 nM and 2.8 ± 0.3 μM, indicating that NBMPR strongly inhibits ENT1-mediated nucleoside transport but weakly inhibits ENT2. Our results demonstrated that dipyridamole, but not NBMPR, inhibited the anti-proliferation action of nucleosides. This suggests that ENT2, which is weakly inhibited by NBMPR, may be involved in the cancer cell growth inhibitory effects of guanosine and 2’-deoxyguanosine. Additionally, previous research by Yamamura et al. [43] has reported the involvement of ENTs in the transport of deoxynucleosides.

We observed a significant increase in DNA content over time in control cells, whereas this increase was not evident in guanosine-treated cells. Additionally, treatment with guanosine, 2’-deoxyguanosine, or DNA hydrolysate resulted in a reduction in the number of BrdU-positive cells compared to control cells. These results suggest that guanosine or 2’-deoxyguanosine hinders the transition of EAT cells from the G1 phase to the S phase, consequently inhibiting DNA synthesis and cell proliferation. Subsequently, we explored the impact of nucleosides on the gene expression of C/EBPβ in EAT cells, as C/EBPβ has been reported to play a crucial role in the proliferation, differentiation, cell cycle, and metabolism of various cells [23–25], particularly in cervical cancer. Recent findings by Long et al. indicated that C/EBPβ decreased cervical tissues, and overexpression of the C/EBPβ gene in cervical cancer cells could inhibit proliferation, invasion, and migration [16]. Additionally, Buck et al. demonstrated that C/EBPβ arrests the cell cycle at the G1/S checkpoint [25]. In our study, we discovered that guanosine and 2’-deoxyguanosine, but not adenosine, increased the gene expression levels of C/EBPβ. Furthermore, we conducted an *in situ* immunofluorescence analysis to examine the intracellular localization of C/EBPβ after the addition of guanosine and 2’-deoxyguanosine, observing the diffusion and presence of C/EBPβ in the nucleus. These findings suggest that guanosine and 2’-deoxyguanosine treatments may positively influence the gene expression of C/EBPβ in EAT cells, potentially impacting cellular processes regulated by C/EBPβ. However, further studies may be necessary to fully comprehend the underlying mechanisms and significance of these observations.

## Conclusions

Our study provides evidence that nucleoside transporters, particularly ENT2, may play a role in the anti-proliferative effects of guanosine and 2’-deoxyguanosine. These nucleosides suppress DNA synthesis and cell proliferation, possibly by interfering with the transition of cells from the G1 phase to the S phase via the activation of C/EBPβ. Our study suggests the involvement of these nucleosides in the regulation of gene expression within the nucleus. This finding is particularly intriguing and warrants further investigation. Our investigation also hints at the potential involvement of these nucleosides in the modulation of gene expression within the cellular nucleus. This discovery is particularly intriguing, prompting the need for further in-depth exploration. Subsequent research endeavors should focus on elucidating the precise molecular mechanisms underpinning these effects, and concurrently, investigating the potential applications of guanosine and 2’-deoxyguanosine in the realm of cancer prevention.

## Supporting information

S1 File (DOCX)
