# Supplementary material for: Suppression of Ehrlich ascites tumor cell proliferation via G1 arrest induced by dietary nucleic acid-derived nucleosides
Source: PLoS One. 2024 Jul 18;19(7):e0305775. doi: 10.1371/journal.pone.0305775 (PMC11257241; doi:10.1371/journal.pone.0305775)
Supplement: S1 File — (DOCX) [file pone.0305775.s001.docx]

Fig. 2. Effect of DNA and RNA on body weight in EAT cell-bearing mice.

Mice were orally administered DNA or RNA (0.05 mg/g body weight) every 2 days for 10 days and then intraperitoneally administered 500 μl of EAT cell suspension adjusted to 0.5 x 10^6^ cells/ml with PBS. After the administration of EAT cells, DNA or RNA was orally administered every day. Data are presented as mean ± SE (n=5-6).

The values of means, standard error (SE) used to build graph of Fig. 2.

| The body weights (g) | | | | | | | | | | | |
| --- | --- | --- | --- | --- | --- | --- | --- | --- | --- | --- | --- |
|  | Days | 0 | 3 | 7 | 10 | 14 | 17 | 21 | 23 | 24 | 25 |
| Control | Mean | 31.38 | 34.23 | 36.21 | 37.38 | 38.31 | 38.68 | 39.74 | 42.22 | 43.55 | 44.94 |
|  | SE | 0.51 | 0.68 | 0.97 | 1.14 | 1.18 | 1.27 | 1.63 | 1.91 | 1.32 | 1.45 |
| DNA | Mean | 31.56 | 34.21 | 35.68 | 37.12 | 36.75 | 37.47 | 37.19 | 37.77 | 39.15 | 39.80 |
|  | SE | 0.28 | 0.47 | 0.59 | 0.61 | 0.63 | 0.72 | 0.89 | 1.16 | 1.67 | 1.83 |
| RNA | Mean | 31.43 | 34.04 | 35.97 | 37.12 | 37.48 | 38.59 | 39.05 | 39.22 | 40.11 | 40.78 |
|  | SE | 0.42 | 0.59 | 0.62 | 0.85 | 1.23 | 0.91 | 0.86 | 0.89 | 0.88 | 0.76 |

Fig. 3. Effect of DNA and RNA on the volume of ascites fluid in EAT cell-bearing mice.

(A) shows the macroscopic figure. The colors attached to the mice were intended for distinguishing between mice within the housing cage, and not for distinguishing between groups. (B) presents the volume of ascites. The mice were orally administered 200 μl of ultrapure water containing 0.05 mg DNA or RNA per gram of body weight as samples. Ten days after the experiment started, 500 μl of EAT cell suspension adjusted to 1.0×10^5^ cells/ml with PBS was intraperitoneally administered to the mice, with the samples continuing to be administered orally on a daily basis. Twenty-five days after the start of the experiment, the mice were dissected under deep isoflurane inhalation anesthesia to minimize pain, and the volume of ascites was measured. The data are shown as mean ± SE (n=5-6) and are considered significantly different relative to the control (**p < 0.01 and *p < 0.05).

The values of means, standard error (SE) used to build graph of Fig. 3 (B).

|  | Ascites volume (ml) | |
| --- | --- | --- |
|  | Mean | SE |
| Control | 7.78 | 0.11 |
| DNA | 2.65 | 0.04 |
| RNA | 1.80 | 0.18 |

Fig. 4. Effect of RNA and DNA on the viability and number of EAT cells.

(A and B) EAT cells were cultured with various concentrations of RNA (0−400 μg/ml) for 24 h. (C and D) EAT cells were cultured with various concentrations of DNA (0−400 μg/ml) for 24 h. The viability (A and C) and number (B and D) of EAT cells were assessed using a trypan blue assay. Data are presented as mean ± SD (n=5). The data were found to be significantly different from the control (**p < 0.01 and *p < 0.05).

The values of means, standard deviation (SD) used to build graph of Fig. 4 (A, B).

|  | Cell viability  (% of Control) | | Cell number  (x10^6^ cells/ml) | |
| --- | --- | --- | --- | --- |
|  | Mean | SD | Mean | SD |
| Control | 100.0 | 1.0 | 1.793 | 0.213 |
| 50 μg/ml RNA | 101.8 | 0.8 | 1.390 | 0.251 |
| 100 μg/ml RNA | 100.2 | 3.2 | 1.377 | 0.206 |
| 200 μg/ml RNA | 99.4 | 3.0 | 1.263 | 0.225 |
| 400 μg/ml RNA | 98.3 | 2.8 | 1.250 | 0.250 |

The values of means, standard deviation (SD) used to build graph of Fig. 4 (C, D).

|  | Cell viability  (% of Control) | | Cell number  (x10^6^ cells/ml) | |
| --- | --- | --- | --- | --- |
|  | Mean | SD | Mean | SD |
| Control | 100.0 | 0.8 | 1.790 | 0.126 |
| 50 μg/ml DNA | 101.8 | 2.7 | 1.760 | 0.260 |
| 100 μg/ml DNA | 100.2 | 0.4 | 1.843 | 0.211 |
| 200 μg/ml DNA | 99.4 | 0.8 | 1.930 | 0.489 |
| 400 μg/ml DNA | 98.3 | 1.6 | 1.683 | 0.292 |

Fig. 5. Effect of DNA hydrolysate on the viability and number of EAT cells.

EAT cells were cultured with various concentrations of DNA hydrolysate (0−400 μg/ml) for 24 h. The viability (A) and number (B) of EAT cells were assessed using a trypan blue assay. Data are presented as mean ± SD (n=5). The data were found to be significantly different from the control (**p < 0.01 and *p < 0.05).

The values of means, standard deviation (SD) used to build graph of Fig. 5 (A, B).

|  | Cell viability  (% of Control) | | Cell number  (x10^6^ cells/ml) | |
| --- | --- | --- | --- | --- |
| Control | Mean | SD | Mean | SD |
| 50 μg/ml DNA hydrolysate | 100.0 | 0.7 | 1.728 | 0.031 |
| 100 μg/ml DNA hydrolysate | 99.6 | 0.9 | 1.698 | 0.031 |
| 200 μg/ml DNA hydrolysate | 100.0 | 0.7 | 1.680 | 0.019 |
| 400 μg/ml DNA hydrolysate | 100.0 | 0.8 | 1.536 | 0.022 |

Fig. 6. Effect of RNA and DNA hydrolysate on the cell number of 3T3-L1 cells.

3T3-L1 cells were cultured with 400 and 800 μg/ml of RNA or DNA hydrolysate for 24 h. The cell number was assessed using a neutral red assay. Data are presented as mean ± SD (n=5).

The values of means, standard deviation (SD) used to build graph of Fig. 5 (A, B).

|  | Cell number  (% of Control) | |
| --- | --- | --- |
|  | Mean | SD |
| Control | 100.0 | 3.6 |
| 400 μg/ml DNA hydrolysate | 100.0 | 2.5 |
| 800 μg/ml DNA hydrolysate | 95.7 | 2.6 |
| 400 μg/ml RNA | 96.7 | 1.1 |
| 800 μg/ml RNA | 96.1 | 1.8 |

Fig. 7. Effect of nucleoside transporter inhibitor on the cell number of RNA and DNA hydrolysate-treated EAT cells.

EAT cells were cultured with 200 μg/ml of DNA hydrolysate or RNA and with or without 10 μM of dipyridamole, an inhibitor of nucleoside transporter, for 24 h. The cell number was assessed using a trypan blue assay. Data are presented as mean ± SD (n=5). The data were found to be significantly different from the control (**p < 0.01).

The values of means, standard deviation (SD) used to build graph of Fig. 7.

|  | Cell number  (x10^6^ cells/ml) | |
| --- | --- | --- |
|  | Mean | SD |
| Control | 1.778 | 0.105 |
| 10 μM Dipyridamole | 1.667 | 0.121 |
| 200 μg/ml DNA hydrolysate | 1.170 | 0.153 |
| 200 μg/ml DNA hydrolysate +10 μM Dipyridamole | 1.628 | 0.182 |
| 200 μg/ml RNA | 1.202 | 0.086 |
| 200 μg/ml RNA +10 uM Dipyridamole | 1.533 | 0.079 |

Fig. 8. Effect of various nucleosides on the cell number of EAT cells.

EAT cells were cultured with (A) 100 μM of adenosine, guanosine, uridine, or cytidine, and (B) 100 μM guanosine or 2-deoxyguanosine for 24 h. The cell number was assessed using a trypan blue assay. Data are presented as mean ± SD (n=6). The data were found to be significantly different from the control (**p < 0.01).

The values of means, standard deviation (SD) used to build graph of Fig. 8 (A).

|  | Cell number  (x10^6^ cells/ml) | |
| --- | --- | --- |
|  | Mean | SD |
| Control | 1.672 | 0.102 |
| 100 μM Adenosine | 1.532 | 0.117 |
| 100 μM Guanosine | 1.307 | 0.102 |
| 100 μM Uridine | 1.74 | 0.102 |
| 100 μM Cytidine | 1.761 | 0.111 |

The values of means, standard deviation (SD) used to build graph of Fig. 8 (B).

|  | Cell number  (x10^6^ cells/ml) | |
| --- | --- | --- |
|  | Mean | SD |
| Control | 1.62 | 0.08 |
| 100 μM Guanosine | 1.02 | 0.10 |
| 100 μM 2’-Deoxyguanosine | 0.99 | 0.12 |

Fig. 9. Effect of nucleoside transporter inhibitor on the cell number of RNA and DNA hydrolysate-treated EAT cells.

EAT cells were cultured with 100 μM guanosine or 2’-deoxyguanosine, with or without 10 μM of dipyridamole, an inhibitor of nucleoside transporter, for 24 h. The cell number was assessed using a trypan blue assay. Data are presented as mean ± SD (n=6). The data were found to be significantly different from the control (**p < 0.01).

The values of means, standard deviation (SD) used to build graph of Fig. 9.

|  | Cell number  (x10^6^ cells/ml) | |
| --- | --- | --- |
|  | Mean | SD |
| Control | 1.72 | 0.07 |
| 10 μM Dipyridamole | 1.51 | 0.06 |
| 100 μM Guanosine | 1.32 | 0.14 |
| 100 μM Guanosine + 10 μM Dipyridamole | 1.53 | 0.13 |
| 100 μM 2’-Deoxyguanosine | 1.25 | 0.13 |
| 100 μM 2’-Deoxyguanosine + 10 μM Dipyridamole | 1.57 | 0.17 |

Fig. 10. Effect of NBMPR, a nucleoside transporter inhibitor, on the cell number of RNA and DNA hydrolysate-treated EAT cells.

EAT cells were cultured with 100 μM guanosine with or without 0.5 μM of NBMPR, an inhibitor of nucleoside transporter, for 24 h. The cell number was assessed using a trypan blue assay. Data are presented as mean ± SD (n=5).

The values of means, standard deviation (SD) used to build graph of Fig. 10.

|  | Cell number  (x10^6^ cells/ml) | |
| --- | --- | --- |
|  | Mean | SD |
| Control | 1.62 | 0.08 |
| 100 μM Guanosine | 1.02 | 0.10 |
| 100 μM Guanosine +0.5 μM NBMPR | 0.91 | 0.17 |
| 0.5 μM NBMPR | 1.44 | 0.19 |

Fig. 11. Effect of guanosine on DNA content of EAT cells. EAT cells were cultured with 100 μM guanosine for 4 - 24 h.

The DNA contents of EAT cells were measured using the Burton procedure. Data are presented as mean ± SD (n=6). The data were found to be significantly different from the control (***p < 0.001).

The values of means, standard deviation (SD) used to build graph of Fig. 11.

|  |  | DNA content (μg) | | | |
| --- | --- | --- | --- | --- | --- |
|  | Time (h) | 4 | 8 | 12 | 24 |
| Control | Mean | 29.063 | 31.875 | 33.938 | 48.188 |
|  | SD | 1.972 | 0.612 | 0.375 | 2.401 |
| 100 μM Guanosine | Mean | 28.125 | 30.750 | 32.250 | 32.438 |
|  | SD | 1.061 | 0.968 | 0.968 | 2.695 |

Fig. 12. Effect of guanosine, 2-deoxyguanosine, or DNA hydrolysate on DNA synthesis of EAT cells.

EAT cells were cultured with BrdU (100 μM) and guanosine, 2-deoxyguanosine, or DNA hydrolysate for 24 h. DNA synthesis was assessed using the BrdU assay, and immunostaining images show BrdU-positive cells. The percentage of BrdU-positive cells was calculated by dividing the BrdU-positive cell number by the total cell number. Data are presented as mean ± SD (n=5). The data were found to be significantly different from the control (**p < 0.01).

The values of means, standard deviation (SD) used to build graph of Fig. 12.

|  | BrdU positive cells (%) | |
| --- | --- | --- |
|  | Mean | SD |
| Control | 89.8 | 6.0 |
| 100 μM Guanosine | 64.6 | 10.7 |
| 100 μM 2’-Deoxyguanosine | 64.3 | 1.9 |
| 200 μg/ml DNA hydrolysate | 47.6 | 9.6 |
| 400 μg/ml DNA hydrolysate | 52.4 | 5.6 |

Fig. 13. Effect of guanosine, 2-deoxyguanosine, or DNA hydrolysate on cell cycle progression of EAT cells.

EAT cells were cultured with guanosine, 2-deoxyguanosine, or DNA hydrolysate for 24 h. Cell cycle was analyzed using the Muse^Ⓡ^ Cell Analyzer. (A) Histograms of DNA content show the distribution of cell cycle phases (G0/G1, S, and G2/M) of EAT cells. (B) The ratio of the cell cycle stages of EAT cells was analyzed. Data are presented as mean ± SD (n = 4). The data were found to be significantly different from the control (**p < 0.01).

The values of means, standard deviation (SD) used to build graph of Fig. 13 (B).

|  | % of total cells | | | | | |
| --- | --- | --- | --- | --- | --- | --- |
|  | G0/G1 | | S | | G2/M | |
|  | Mean | SD | Mean | SD | Mean | SD |
| Control | 39.4 | 1.2 | 30.6 | 3.3 | 29.6 | 2.7 |
| 100 μM Guanosine | 45.9 | 1.4 | 27.2 | 1.7 | 26.6 | 0.5 |
| 100 μM 2’-Deoxyguanosine | 44.0 | 1.9 | 29.6 | 0.9 | 26.1 | 2.2 |
| 200 μg/ml DNA hydrolysate | 43.4 | 0.7 | 27.1 | 2.0 | 29.3 | 2.2 |

Fig. 14. Effect of guanosine and dipyridamole, a nucleoside transporter inhibitor, on DNA synthesis of EAT cells.

EATC were cultured with BrdU (100 μM) and dipyridamole (10 μM) for 24 h. DNA synthesis was assessed using the BrdU assay. Immunostaining images show BrdU-positive cells. The percentage of BrdU-positive cells was calculated by BrdU-positive cell number relative to the total cell number. Data are presented as mean ± SD (n = 5). The data were considered significantly different relative to the control (**p < 0.01).

The values of means, standard deviation (SD) used to build graph of Fig. 14.

|  | BrdU positive cells (%) | |
| --- | --- | --- |
|  | Mean | SD |
| Control | 92.4 | 8.4 |
| 100 μM Guanosine | 31.6 | 3.7 |
| 100 μM Guanosine + 10 μM Dipyridamole | 83.7 | 5.3 |

Fig. 15. Effect of guanosine, 2’-deoxyguanosine or adenosine on the gene expression of C/EBPβ.

mRNA expression was analyzed by qRT-PCR 24 h after incubation of guanosine-, 2’-deoxyguanosine- or adenosine-treated EAT cells. Data are presented as mean ± SD (n=3).

The values of means, standard deviation (SD) used to build graph of Fig. 15.

|  | C/EBPβ mRNA level (Relative to control) | |
| --- | --- | --- |
|  | Mean | SD |
| Control | 1.00 | 0.2 |
| 100 μM Guanosine | 1.53 | 0.24 |
| 100 μM 2’-Deoxyguanosine | 1.57 | 0.47 |
| 100 μM Adenosine | 1.01 | 0.33 |
